# A new, exceptionally preserved juvenile specimen of *Eusaurosphargis dalsassoi* (Diapsida) and implications for Mesozoic marine diapsid phylogeny

**DOI:** 10.1038/s41598-017-04514-x

**Published:** 2017-06-30

**Authors:** Torsten M. Scheyer, James M. Neenan, Timea Bodogan, Heinz Furrer, Christian Obrist, Mathieu Plamondon

**Affiliations:** 1 Universität Zürich, Paläontologisches Institut und Museum, Karl Schmid-Strasse 4, CH-8006 Zurich, Switzerland; 2 Oxford University Museum of Natural History, Parks Road, Oxford, OX1 3PW UK; 3 Erliackerweg 8, Rickenbach, BL 4462 Switzerland; 4 Empa, Swiss Federal Laboratories for Materials Science and Technology, Center for X-ray Analytics, Überlandstrasse 129, CH-8600 Dübendorf, Switzerland

## Abstract

Recently it was suggested that the phylogenetic clustering of Mesozoic marine reptile lineages, such as thalattosaurs, the very successful fish-shaped ichthyosaurs and sauropterygians (including plesiosaurs), among others, in a so-called ‘superclade’ is an artefact linked to convergent evolution of morphological characters associated with a shared marine lifestyle. Accordingly, partial ‘un-scoring’ of the problematic phylogenetic characters was proposed. Here we report a new, exceptionally preserved and mostly articulated juvenile skeleton of the diapsid reptile, *Eusaurosphargis dalsassoi*, a species previously recovered within the marine reptile ‘superclade’, for which we now provide a revised diagnosis. Using micro-computed tomography, we show that besides having a deep skull with a short and broad rostrum, the most outstanding feature of the new specimen is extensive, complex body armour, mostly preserved *in situ*, along its vertebrae, ribs, and forelimbs, as well as a row of flat, keeled ventrolateral osteoderms associated with the gastralia. As a whole, the anatomical features support an essentially terrestrial lifestyle of the animal. A review of the proposed partial character ‘un-scoring’ using three published data matrices indicate that this approach is flawed and should be avoided, and that within the marine reptile ‘superclade’ *E*. *dalsassoi* potentially is the sister taxon of Sauropterygia.

## Introduction

The new, complete and less than 20 cm long skeleton of the small diapsid *Eusaurosphargis dalsassoi* Nosotti and Rieppel, 2003 (PIMUZ A/III 4380, Palaeontological Institute and Museum of the University of Zurich, Switzerland) was discovered in the Middle Triassic Prosanto Formation, exposed in proximity to Ducanfurgga, a locality near Davos, Canton Grisons, in the Eastern Swiss Alps. This area recently gained importance as another Swiss locality yielding outstanding Middle Triassic fossils which, in some respects, are similar to those known from the slightly older Besano and Meride Formations of Monte San Giorgio in Canton Ticino and Northern Italy^1–4^. Within the fauna, *E*. *dalsassoi* is a new and very rare element, previously only known by few disarticulated specimens from Besano, Italy (Besano Formation, Anisian/Ladinian boundary^5^, and Winterswijk, The Netherlands (Vossenfeld Formation, Anisian^6–8^).

In the phylogenetic analysis accompanying the original description, *Eusaurosphargis dalsassoi* was recovered as sister taxon to *Helveticosaurus zollingeri* ^5^, a large marine reptile from the Besano Formation (Anisian/Ladinian) of Monte San Giorgio, southern Switzerland^9, 10^. The paedomorphic appearance of morphological features (e.g., reduced size of pectoral and pelvic girdle elements; lack of ossified epiphyses of stylopodial and zeugopodial elements, retention of open neurocentral sutures) of *H*. *zollingeri* indicated an overall aquatic lifestyle for this animal, but terrestrial excursions were not ruled out^10^. In the case of the much smaller holotype of *E*. *dalsassoi*, the assessment of habitat preference was less clear. Based on both anatomical features (such as neurocentral suture closure in the holotype) and the extreme scarcity of fossils, a terrestrial lifestyle was considered possible in *E*. *dalsassoi* ^5^. Subsequent analyses either recovered a sister group relationship of *H*. *zollingeri* and *E*. *dalsassoi* ^11, 12^, as successive sister taxon to a group including *Helveticosaurus*, Ichthyopterygia, *Sinosaurosphargis* and Sauropterygia^13^, or a sister grouping of *Eusaurosphargis* with *Hanosaurus hupehensis* ^14, 15^, an enigmatic reptile from the Jialingjiang Formation (Spathian/Anisian?) of China. *Helveticosaurus* was recovered as sister to (*Eusaurosphargis*, *Hanosaurus*) in a single analysis when several poorly-known Chinese marine reptile taxa were excluded from the dataset^15^. In all these analyses, however, *E*. *dalsassoi* was found close to or nested well within the major aquatic groups of the Mesozoic reptiles, including the recently erected Saurosphargidae^11, 12^.

**Figure 1:**
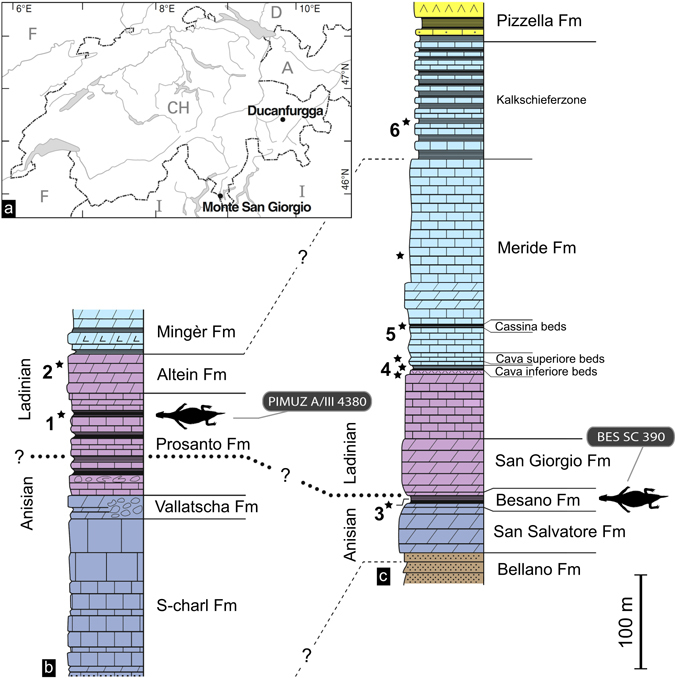
Locality and stratigraphic position of the alpine specimens of *Eusaurosphargis dalsassoi* (PIMUZ A/III 4380 and holotype specimen BES SC 390). (**a**) Map showing the Ducanfurgga locality relative to the World Heritage vertebrate site of Monte San Giorgio. (**b**) Correlation of the Middle Triassic section at Ducanfurgga (Upper Austroalpine Silvretta Nappe, south-eastern Switzerland) with that of (**c**) Monte San Giorgio (southern Alps, southern Switzerland). Reproduced from Fig. 1 of Fraser and Furrer (2013)^3^, with permission of the Swiss Geological Society. Relevant ages according to U-Pb zircon dating of volcaniclastic layers are 240.91 ± 0.26 Ma^19^ (1*), 239.89 ± 0.21 Ma^19^ (2*), 242.1 ± 0.6 Ma^55^ (3*), 241.07 ± 0.13 Ma^20^ (4*), 240.63 ± 0.13 Ma^20^ (5*), and 239.51 ± 0.15 Ma^20^ (6*).

At an altitude of 2600 to 2800 metres, the Prosanto Formation is exposed as a 120 m thick sequence of dark limestones, shales and dolomites (Fig. 1) at a mountain crest southeast of the locality Ducanfurgga near Davos (Canton Grisons, southeastern Switzerland). Within the tectonically deformed sediments of the Austroalpine Silvretta Nappe, the sediments of the Prosanto Formation were deposited in a small shallow, stratified intra-platform basin with sub-euxinic deeper bottom waters, which extended for about 20 km^1, 16^. Fishes and reptiles inhabited the shallower waters of the basin margins and the upper part of the water column; whereas oxygen depletion in deeper waters prevented scavenging of the vertebrate carcasses, thus enabling the exquisite preservation of the fossil remains within the fine-grained carbonate and siliciclastic sediments. A sporadic input from islands and land masses nearby is indicated by the presence of terrestrial faunal elements (insects; remains of the rauisuchian, *Ticinosuchus*, and of the tanystropheid protorosaur, *Macrocnemus*) and land plants^2, 3, 17, 18^.

The lithostratigraphic similarities between the Prosanto Formation and the Middle Triassic formations of Monte San Giorgio (Fig. 1) were later corroborated by U-Pb zircon ages^19^ from volcanic ash layers and tuffs in the fossiliferous beds of the Upper Prosanto Formation (240.91 ± 0.26 Ma) and the overlying Altein Formation (239.89 ± 0.21 Ma). As such, the horizon from which PIMUZ A/III 4380 derives is correlated with the Early Ladinian vertebrate-bearing horizons (Cava inferiore and Cava superiore beds, *P*. *gredleri* Zone) of the Lower Meride Limestone^3, 19, 20^.

**Figure 2:**
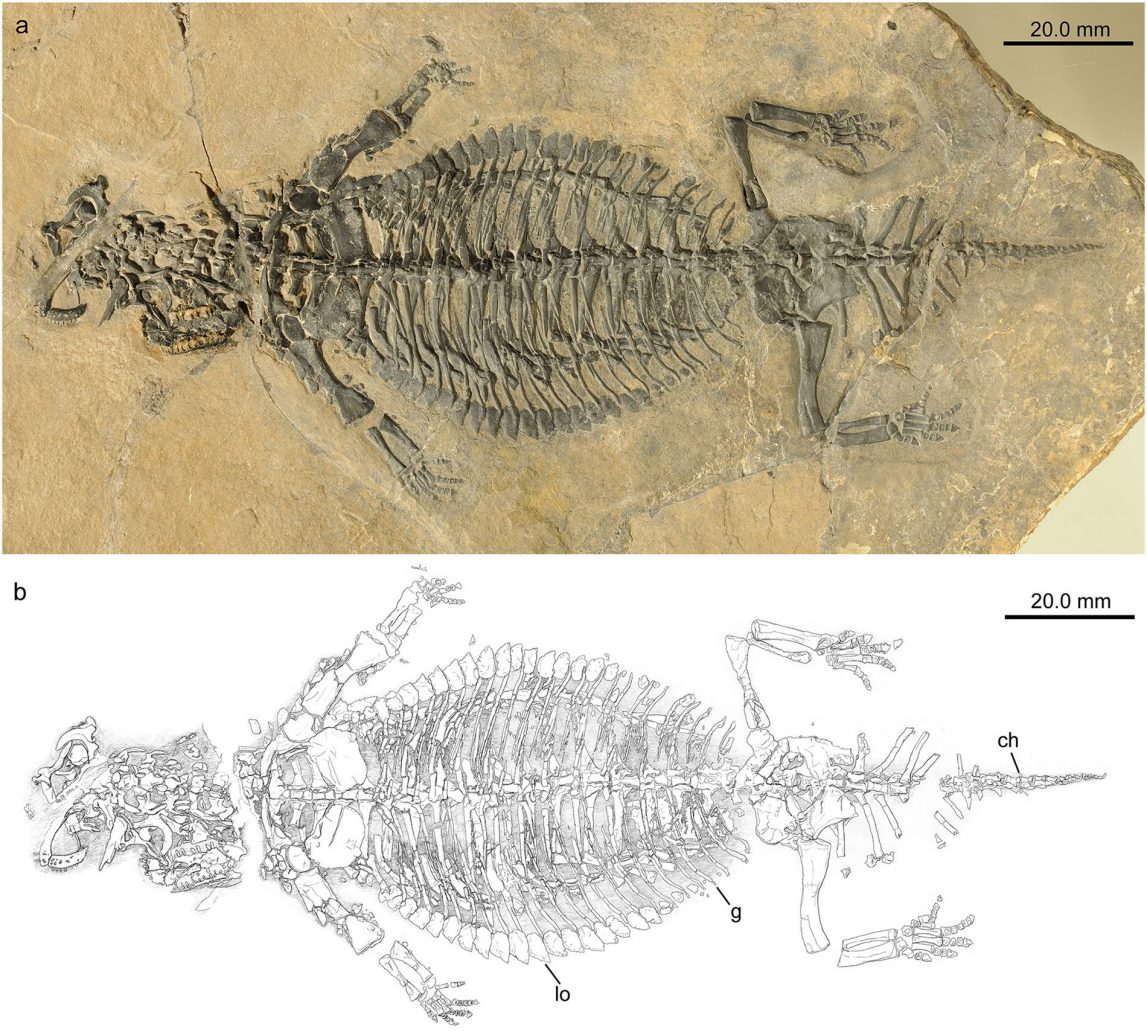
*Eusaurosphargis dalsassoi* (PIMUZ A/III 4380) in ventral view. (**a**) Photograph. (**b**) Interpretative drawing of skeleton. Abbreviations used in the figure are chevron (ch), gastralia (g), and lateral osteoderm (lo).

**Figure 3:**
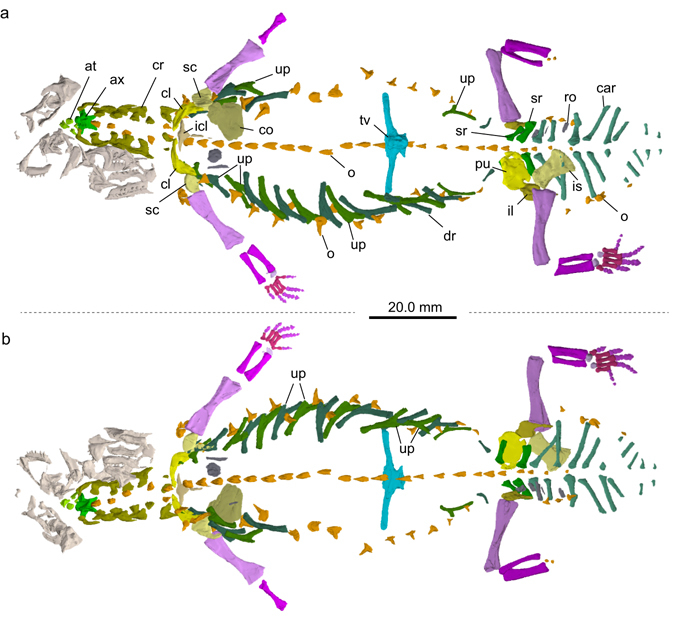
Virtual reconstruction of *Eusaurosphargis dalsassoi* (PIMUZ A/III 4380) with focus on postcranial elements. (**a**) Ventral and (**b**) dorsal view. Note that due to preservational aspects, not all bones of the skeleton were reconstructed. Unidentified bones are shown in dark grey and skull bones in light grey. Abbreviations used in the figure are atlas complex (at), axis (ax), caudal rib (car), clavicle (cl), coracoid (co), cervical rib (cr), dorsal (thoracic) rib (dr), interclavicle (icl), ilium (il), ischium (is), osteoderm (o), pubis (pu), rectangular osteoderm (ro), scapula (sc), sacral rib (sr), thoracic vertebra (tv), and uncinate process (up).

**Figure 4:**
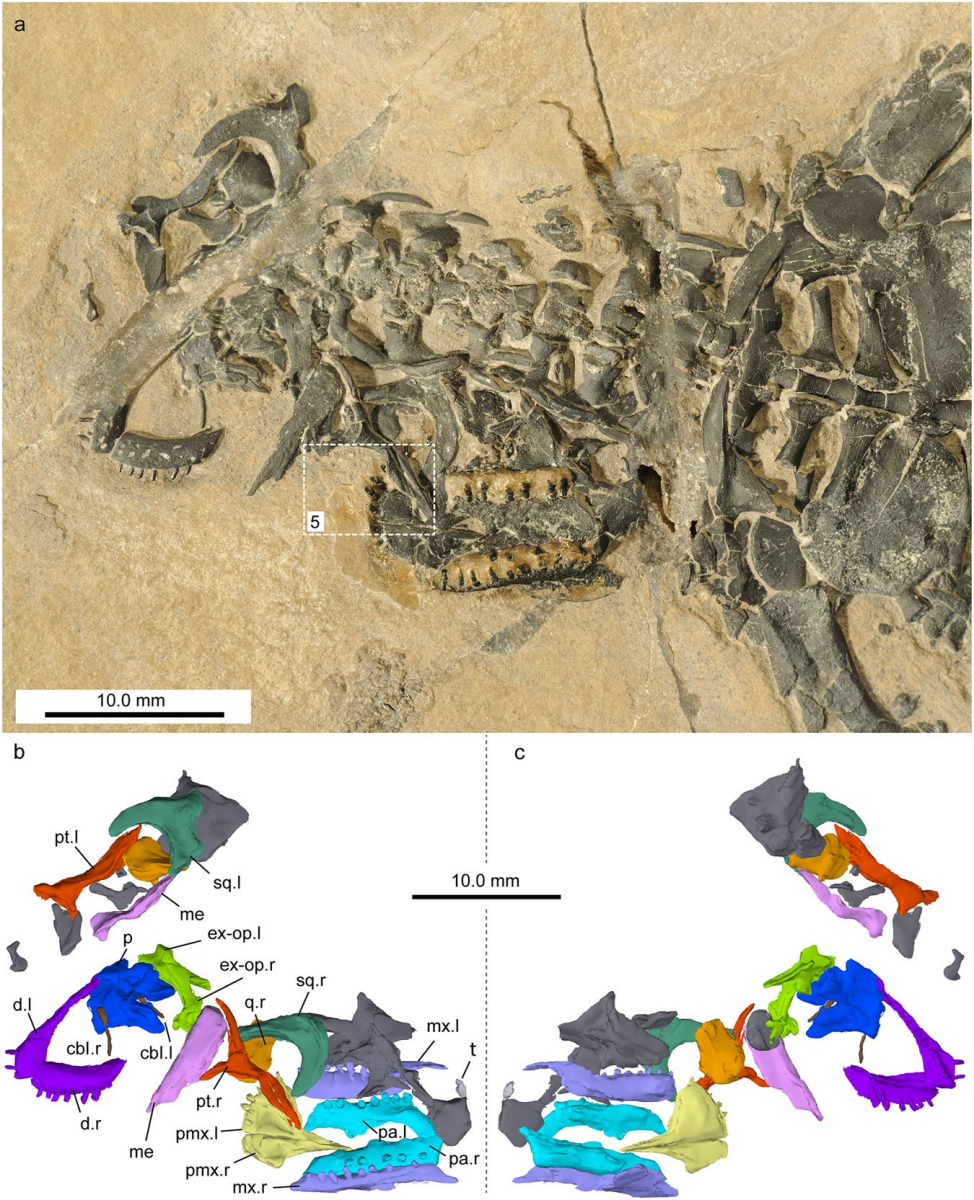
Skull and lower jaw elements of *Eusaurosphargis dalsassoi* (PIMUZ A/III 4380) in ventral view. (**a**) Photograph. White rectangle indicates section of skull shown in Fig. 5. (**b**,**c**) Virtual reconstruction of the cranial bones in ventral (**b**) and dorsal (**c**) view. Unidentified bones are shown in dark grey. Note that when possible the left (.l) and right (.r) side is indicated for the identified elements. Abbreviations used in the figure are ceratobranchial I (cbI), dentary (d), exoccipital-opisthotic (ex-op), mandible element (me), maxilla (mx), parietals (p), palatine (pa), premaxilla (pmx), pterygoid (pt), quadrate (q), squamosal (sq), and tooth (t).

Herein we provide a detailed description of the new fossil material assignable to *Eusaurosphargis dalsassoi*, using classical osteology and micro-computed tomography (Figs 2–4), revise the diagnosis of the taxon, as well as elucidate its phylogenetic position based on this revision. In addition, the lifestyle of the species is re-assessed. Furthermore, the treatment of characters for phylogenetic analyses, which are potentially influenced by a convergent marine lifestyle, is evaluated.

## Results

### Systematic Palaeontology

Diapsida Osborn, 1903^21^.

*Eusaurosphargis dalsassoi* Nosotti and Rieppel, 2003.

### Holotype

BES SC 390 (Palaeontological Collection of the Museo Civico di Storia Naturale di Milano, Italy), an associated but disarticulated specimen from the Besano Formation (242 Ma, Anisian-Ladinian, Middle Triassic) of Besano, Lombardy, Italy.

### Referred specimen

PIMUZ A/III 4380, an almost completely articulated skeleton preserved in ventral view (Fig. 2a).

### Stratigraphy and locality

Upper Prosanto Formation (bed no. 1; fossil found on bottom side of a limestone slab); 241 Ma, early Ladinian, Middle Triassic of Ducanfurgga locality no. 4, south of Davos, Canton Grisons (Graubünden), south-eastern Switzerland.

### Description

PIMUZ A/III 4380 represents a complete skeleton of a small diapsid of less than 200 mm body length. The specimen shows exquisite preservation and is almost fully articulated; the skull, however, together with the mandibles has disassembled and is now lying adjacent to the neck vertebrae anterior to the pectoral girdle elements. The rest of the body, from the 1^st^ cervical vertebra to the terminal caudal vertebral centrum, measures about 156 mm in length (detailed measurements are compiled in Table 1).

**Table 1:** Measurements of PIMUZ A/III 4380 in [mm], rounded to the first decimal place.

Total body length (without skull)	155.6	
Minimum length of cervical region (six cervicals in articulation, without atlas)	23.9	
Length of thoracic region (18 thoracic vertebrae/dorsals)	74.5	
Length of sacral region (2 sacrals)x	7.8	
Length of tail (20 caudals)	49.4	
**Element**	**Right**	**Left**
Humerus length	15.9	15.8
Humerus proximal width	4.4	4.3
Humerus mid-shaft width	2. 4	2.5
Humerus distal width	5.7	5.9
Radius length	9.0	8.1
Ulna length	7.8	7.4
Femur length	17.9	nm
Femur proximal width	5.5	nm
Femur mid-shaft width	2.6	nm
Femur distal width	3.8	3.9
Tibia length	9.1 (broken proximally)	10.4
Fibula length	8.3 (broken proximally)	10.8
Length of autopodium (hand, measured at 2nd digit)	10.5	nm
Length of autopodium (foot, measured at 2nd digit)	13.4	13.5
Length of exposed ceratobranchial I	3.6	3.5
Diameter of ceratobranchial I at head	0.6	0.5
Diameter of ceratobranchial I at mid-shaft	0.2	0.3
Length of premaxilla	~6.5	nm
Length of maxilla	11.1	nm
Coracoid short axis length (longitudinal)	7.9	7.7
Coracoid long axis length (transverse)	10.1	9.8
Interclavicle length	7.8	
Interclavicle width	10.7	
Mid-trunk (13th) vertebral transverse process width	~23.3	
Pubis short axis length (longitudinal)	5.3	
Pubis long axis length (transverse)	9.5	
Ischium long axis length (medio-lateral)	9.5	
Ischium medial expansion	7.1	
Ischium least mid-shaft diameter	3.9	
Ischium lateral expansion	5.0	

Abbreviations: nm = not measurable.

### Revised diagnosis (for genus and species) based on holotype and new material

Small to medium sized diapsid with deep skull and robust lower jaw; skull with relatively small upper temporal fenestrae; posterior margin of skull table straight or only weakly concave; pineal foramen located at level of the anterior margin of the upper temporal fenestrae; double tooth row in upper jaw (one each in the maxilla and palatine); homodont dentition that is at least subthecodont; tooth crowns leaf shaped, with distinct lingual heel; vertebral centra platycoelous or weakly amphicoelous and non-notochordal; cervical and anterior caudal vertebrae with short neural spines; dorsal vertebrae with low neural spine and distinctly elongate transverse processes, located at anterior end of vertebra and trending in an anterolateral direction; prezygapophyses of dorsal vertebrae emerging from a crest extending along the anterior surface of the transverse processes; thoracic ribs characterised by distinct prong- to fan-shaped uncinate processes (convergent to some degree in *Paraplacodus*), anterior thoracic ribs not expanded distally; vertebral column consisting of 7 cervicals, 18 thoracic vertebrae (=dorsals), 2 sacrals and 20 caudals (47 in total); cervical vertebrae carrying robust and interlocking (overlapping) cervical ribs; small round to oblong, oval or elliptical osteoderms, tapering laterally, with irregular margins and pitted surfaces forming lateral row associated with gastralia; rows of conical osteoderms with off-centred apices and broad uneven bases topping the neural spines and ribs (probably associated with the uncinate processes); flat overlapping shingle-like and more round osteoderms covering shoulder girdle as well as forelimbs preaxially and postaxially; humerus with straight shaft, slightly more expanded distally compared to proximal part; radius and ulna slightly curved framing narrow spatium interosseum; shaft of femur slightly sigmoidal, proximally more strongly expanded compared to distal part; humeri and femora carrying well developed, slightly angled epicondylar articulation facets for zeugopodial elements; T-shaped interclavicle large with prominent posterior process; thyroid fenestra between pubis and ischium large; obturator foramen of pubis forming wide and deep notch postero-laterally; ischium with shaft and distal fan-shaped expansion.

### Comparison

PIMUZ A/III 4380 most closely resembles the holotype specimen of *Eusaurosphargis dalsassoi* in outer morphology. Furthermore, following^5^ and contra^11^, we consider “*Saurosphargis volzi*” from the Lower Anisian (lower Muschelkalk) of Gogolin, lower Silesia, a nomen dubium due to a) the loss of the holotype and only specimen and b) the lack of diagnostic characters to be gleaned from the only remaining photographs and original description^22^ (see also discussion in ref. 5).

PIMUZ A/III 4380 shares with placodont sauropterygians the strongly elongated transverse processes of the thoracic vertebrae and the presence of osteoderms, although the arrangement of the latter differs considerably. It differs from Sauropterygia in general, however, in lacking a curved humerus, in having a T-shaped interclavicle in which the posterior process is not reduced or rudimentary, as well as a clavicle that is not applied to the antero-medial surface of the scapula. It further differs from Chinese members of Saurosphargidae Li, Rieppel, Wu, Zhao and Wang, 2011, another group characterised by elongated transverse processes and osteoderms including *Sinosaurosphargis yunguiensis* ^12^, and two species of *Largocephalosaurus* ^11, 23^, in lacking a curved humerus, retracted external nares, a constricted rostrum and a body which is dorsally covered (to a large degree) with small, sutured polygonal osteoderms. In comparison to the saurosphargids, the gastral apparatus is less massively built in PIMUZ A/III 4380 and the median elements are not bifurcated.

### Skull and lower jaw

While the postcranium of PIMUZ A/III 4380 is virtually complete and articulated (Figs 2 and 3; Supplementary Fig. S1), the cranium is mostly disarticulated (Fig. 4; Supplementary Figure S2). Due to this, confident identification of many of the bones cannot be made, despite clarification through μCT scanning. Difficulties of identification are exacerbated by a large crack that runs through the anterior of the specimen which has destroyed portions of several skull bones and, most likely, has obliterated some entirely. Therefore, only elements that can be positively identified or recognised as being paired are described below.

#### Premaxilla

**Figure 5:**
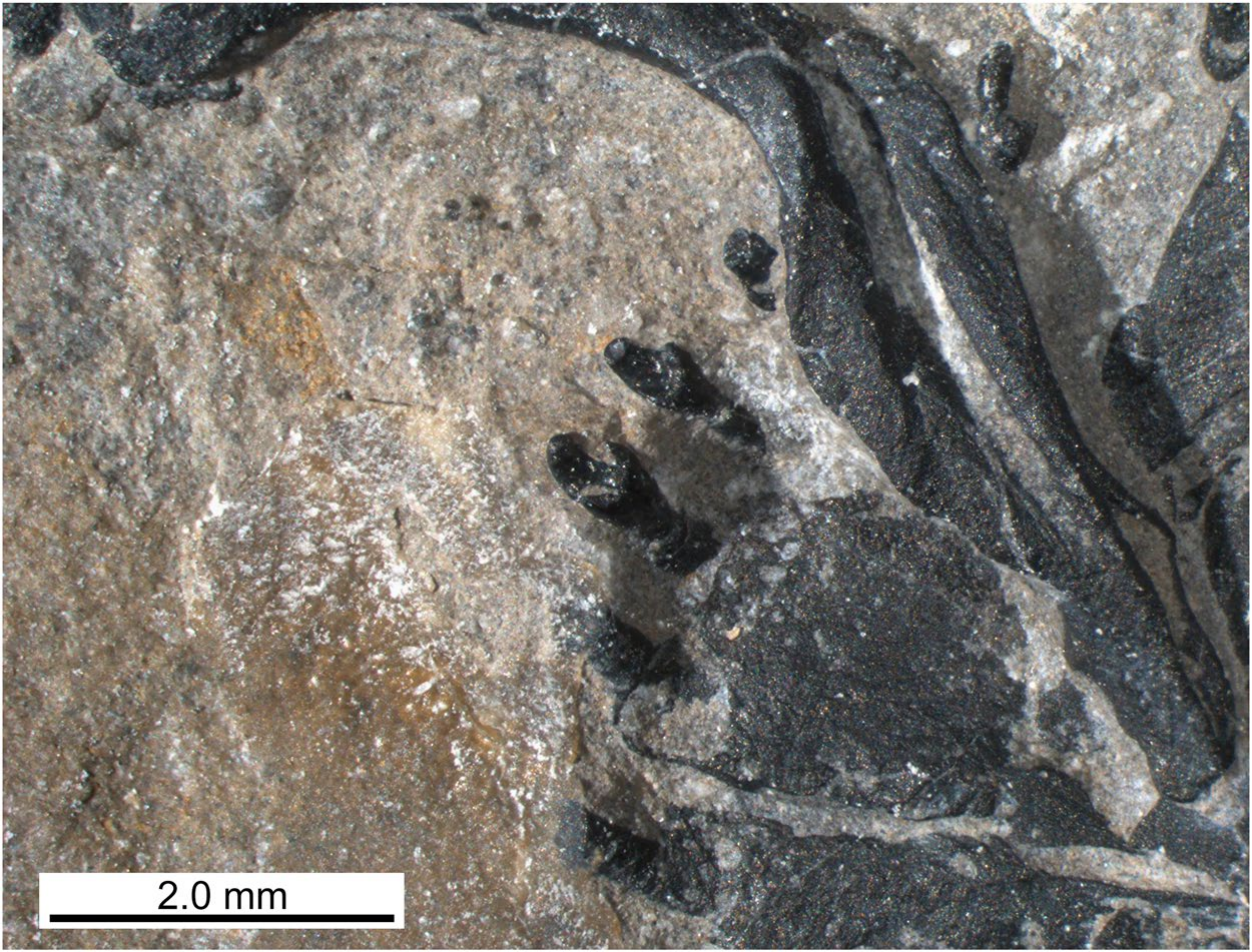
Detail of the premaxillary region of the skull of *Eusaurosphargis dalsassoi* (PIMUZ A/III 4380).

Both premaxillae are present, are articulated with one another, and are exposed in ventral view. The general morphology is very similar to that of the holotype, BES SC 390. Together they form a spatulate rostrum, with a wide, gently curving anterior margin. Each premaxilla has a long, narrow and pointed process which would have extended posteriorly along the medial midline of the skull. The left premaxilla has four teeth preserved *in situ*, with a disarticulated tooth located nearby, indicating that each premaxilla had a minimum of five teeth each. The right premaxilla, however, only has two teeth preserved and is less complete than the left. The premaxillary dentition is better preserved in PIMUZ A/III 4380 than in the holotype, with the tooth crowns exhibiting an unusual, curved, ‘spade-like’ morphology with a round base that becomes narrower in the anteroposterior axis, but not in the mesio-lateral one towards the distal tip of each tooth (Fig. 5).

#### Maxilla

Both maxillae are present, are articulated with the premaxillae and palatines, and are preserved in ventral view. The maxilla is a long, narrow element that remains marginal, and does not progress medially to any great degree. The posterior margin tapers to a narrow termination laterally. The right maxilla is better preserved than the left and has a wide dorsal process at its anterior margin. This may be an artefact of preservation, however, and any posterior dorsal extension may have simply been broken. There are at least eight teeth preserved in both maxillae which are of a similar morphology to those of the premaxilla, albeit more pointed and less ‘spade-like’.

#### Palatine

Both palatines are present, are roughly articulated with the maxillae and premaxillae, and are preserved in ventral orientation. It appears that the palatines met in a medial suture, as no other elements can be identified between them. The palatine is a dorso-ventrally flattened, elongate element that is roughly equal in length to the maxilla. The posterior margin has a slight taper that ends in a posteriorly-orientated point on the medial margin, although in the left palatine, this bends in medially. It is unclear whether this is taphonomic in origin, however. The left and right palatines have at least seven and five preserved teeth respectively. These are organised as a single tooth row, similar to the condition in *Palatodonta* ^14^, and are not considerably different in morphology from those of the maxilla.

#### Pterygoid

The pterygoids are edentulous and are both visible in the specimen, although a portion of the left one is missing due to the crack running through it. The shape of the pterygoid is strikingly similar to the condition seen in many squamates, particularly varanoids (e.g., ref. 24, in that it is a long, narrow, slightly-curved, rod-like element with a distinct lateral process; creating a roughly Y-shaped appearance).

#### Parietal

The parietals are preserved in dorsal view, and appear to be fused (they were described as being unfused in the holotype specimen BES SC 390). Together they form a broad, flat element that contains a small pineal foramen near the anterior margin. The anterolateral margins extend forwards, creating a ‘v-shaped’ embayment which would have articulated with other skull roof bones. The ventral surface has a medial concavity posterior to the pineal foramen, but this does not appear to be concordant with the parietal depressions described in the holotype.

#### Squamosal

Both squamosals are present and clearly visible. The squamosal is a flat, curved, and sickle-shaped element, which most likely formed the posterior margin of the upper temporal fenestra. It differs slightly from the description of the holotype specimen BES SC 390^5^ in that it lacks a significant posterior extension. However the resemblance to the condition seen in some thalattosaur specimens (i.e., of *Askeptosaurus italicus*) is striking^25^, and aided in the identification of this element.

#### Quadrate

Both elements are present and are associated closely with the medial-most portions of the squamosals, indicating a possible articulation at this point. The quadrate is a short element that is approximately twice as long as it is wide. The left element is better preserved than the right, with a tapering ventral process that possibly represents the articulation with the articular.

#### Exoccipital-Opisthotic

Both elements are preserved in close proximity to one another but are disarticulated. There is a prominent and elongated paroccipital process which would have projected laterally. The medial margin is pillar-like and slightly curved, forming the lateral margins of the foramen magnum.

#### Hyoid apparatus

A pair of long, narrow, slightly curved rods, closely associated with the mandible, were identified as the ceratobranchials I. Anteriorly, the bones show a thickened head region, whereas the shaft is of similar thickness throughout.

#### Mandible

Three distinct elements belonging to the mandible can be identified and are preserved in ventral view. The dentaries are articulated, but incomplete, with the right element being broken and the left intersecting the aforementioned crack. The right dentary contains at least six teeth, with only two being present in the left. However, four distinct empty tooth sockets can be identified in the latter, confirming that the dentition of *Eusaurosphargis* is at least subthecodont^5, 26^. These teeth are similar to those of the maxilla and the premaxillary teeth present in the holotype. The lingual edge of the dorsal surface contains a distinct ridge that appears to run the length of the dentary. A series of distinct but small foramina are also present on the ventral surface, probably for vasculature related to the teeth, or possibly for sensory nerves.

The remaining two mandibular elements are likely conglomerates of more than one mandibular bone, possibly including the posterior margins of the dentaries and more posteriorly situated bones such as the angulars. The right element is long and wide, with an anterior taper. The left one, on the other hand, is damaged due to a crack extending through the sediment matrix.

### Axial skeleton

With the exception of the distal tip of the tail all vertebrae in the column are exposed in ventral view (Fig. 2). There are no ventral keels developed in any of the antero-posteriorly expanded cylinder-shaped centra of the vertebral column, nor were there any isolated chevron bones found in PIMUZ A/III 4380 or in the holotype BES SC 390. A single small knoblike bony element at the posteroventral margin of caudal 11 (Fig. 2) might represent a much reduced chevron, in which case the described ‘posteroventral pedicles’ of the caudals in the holotype might actually also be interpreted as being small, reduced or even fused chevrons instead (the bone identified as a chevron bone in the BES SC 390 in Fig. 16 in ref. 5 is herein interpreted as a cervical rib in lateral view). Where exposed, the articular surfaces of the vertebral centra are platycoelous or only slightly amphicoelous and non-notochordal (just visible in cervical 3 and thoracic vertebra 16, but corroborated by μCT scan data). The zygapophyses along the vertebral column are covered by sediment and are thus not visible on the specimen itself. The μCT scan data, however, revealed short prezygapophyses that extend little beyond the anterior margins of the neural arches, as well as postzygapophyses which extend well beyond the posterior margins. Neurocentral sutures between centra and neural arches could neither be seen on the fossil nor studied in the μCT scan.

#### Cervical region

The neck region comprises seven cervical vertebrae in total, six of which, including the axis, are still in articulation (Fig. 4). Directly anterior to the axis four bony elements were recognised in the μCT scan data, which pertain to the now disarticulated atlas complex. These elements include a small angled triangular element, identified as an intercentrum, a larger roundish element which is tentatively identified as a pleurocentrum, as well as two elongated and posteriorly slightly forked elements that constitute the left and right parts of the neural arch. Whether the atlas also carried ribs or not could not be identified in the scan. There is also no indication that the atlas was topped dorsally by an osteoderm. The axis carries an anteroposteriorly elongated neural arch, which is not covered by an osteoderm, whereas cervicals 3 to 7 dorsally carry prominent, conical osteoderms on the neural arches. The μCT scan data of PIMUZ A/III 4380 thus corroborate the general morphology of the cervical vertebrae described in the holotype BES SC 390 (see Fig. 6a in ref. 5), with the exception of the purported ‘triangular neural spine’. This triangular spine is actually a separate conical osteoderm, the rough base of which is about as wide as the neural spine, and the overlying osteoderm also has a characteristic pitted surface texture (isolated and attached osteoderms with similar surface texture are also known from NMNHL RGM 449487 from the Netherlands^27^). The centrum of the axis has a concave anterior and a flat posterior condyle, whereas the centra of cervicals 3 to 7 are platycoelous or slightly amphicoelous. Apart from the axis cervical ribs, the remaining cervical vertebrae have short transverse processes that articulate with massive cervical ribs, whose articular heads are perpendicularly oriented to the long axis of the ribs. All heads of the cervical ribs are dichocephalic; a feature clearly visible on the fossil, but difficult to distinguish in the μCT scan images due to the overlap of bones. Laterally, the rib shafts extend into an anteriorly tapering process and a posterior blade-like portion ending in a ventral short posterior process (a similar morphology was depicted in Fig. 6b in ref. 5 for the holotype BES SC 390; the specimen in Fig. 6c in ref. 5 is broken posteriorly and the anterior process is not shown due to an overlapping bone in the holotype specimen). Although slightly divergent in shape the posterior blade-like portion of the cervical ribs forms a shallow trough which accommodates the anterior process of the following cervical rib. In the axis, the transverse processes and cervical ribs are less massive, the anterior process of the rib is very short and the posterior blade-like part is less expanded in comparison to the other cervical ribs. Cervical ribs 5 and 6 on the left side and cervical ribs 6 and 7 are partially broken where the rock was initially split during excavation. The left cervical rib 7, however, lacks the broadened blade-like part and instead terminates in a single tapering process.

**Figure 6:**
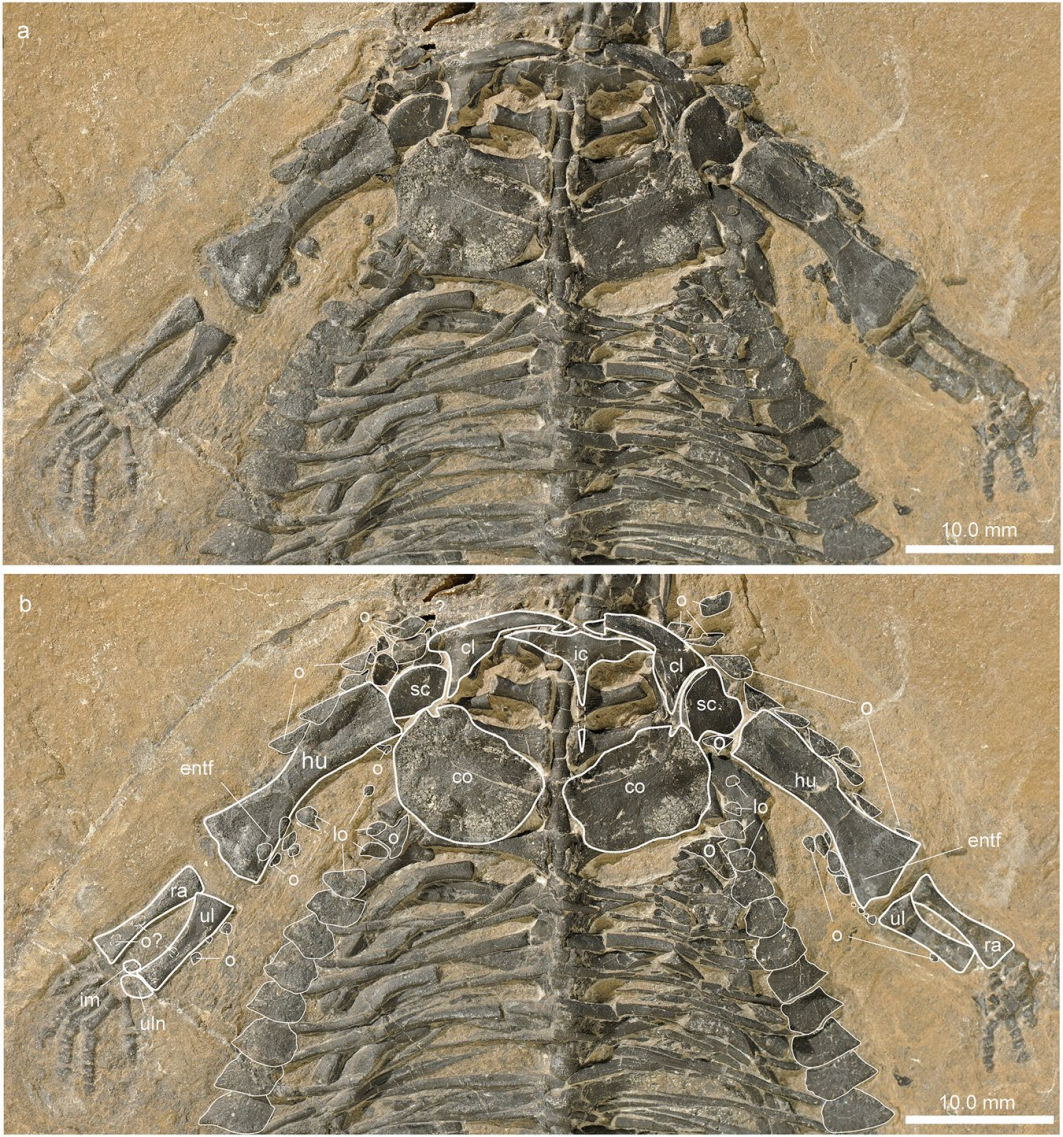
Pectoral and anterior thoracic region of *Eusaurosphargis dalsassoi* (PIMUZ A/III 4380). (**a**) Photograph. (**b**) Photograph overlain by interpretative drawing. Abbreviations used in the figure are clavicle (cl), coracoid (co), entepicondylar foramen (entf), humerus (hu), interclavicle (icl), intermedium (im), lateral osteoderm (lo), osteoderm (o), radius (ra), scapula (sc), ulna (ul), and ulnare (uln).

#### Dorsal region

The thoracic or dorsal series consists of 18 vertebrae in total (Fig. 2a,b), with their overall morphology being accessible mostly in the μCT scan data. The centra and neural arches of the thoracic vertebrae resemble those of the cervicals 3 to 7, but the transverse processes, angled anterolateral from each vertebral centrum, become exceedingly elongated in the mid trunk region. The transverse processes become shorter again in the posterior trunk region, and the last thoracic vertebra, the 25^th^ from anterior, has again quite short transverse processes. Where the transverse processes became detached from the respective centra during fossilisation, a highly rugose and anteroposteriorly expanded articular surface is exposed, which was likely covered by cartilage. This separation is especially visible in the first five thoracic vertebrae.

Each thoracic vertebra has a single osteoderm resting dorsally on its neural arch, as well as a pair of proximo-distally curved or angled monocephalic ribs. The anterior thoracic ribs are not expanded distally, whereas there is a slight expansion in the more posteriorly situated ones. All thoracic ribs carry bony extensions from the rib shaft, so-called uncinate processes, which vary strongly in morphology and orientation throughout the series (Fig. 3; Supplementary Fig. S1). The longest ribs are situated in the mid trunk region of the animal. Besides a strong increase in size, dorsal ribs 1 to 3 all carry an anteriorly extending process, which is a simple tapering prong in the first two ribs and a more flange- or blade like process in the third rib. The dorsal ribs 4 to 13 are longer, distinctly curved or angled, and with a posteriorly oriented, broad and blade-like uncinate process. The uncinate process becomes again narrower and bluntly prong-like in dorsal ribs 14 to 17. The short and slightly curved last dorsal ribs lack an uncinate process.

#### Sacral region

The sacral region is largely covered ventrally by the platy girdle elements, the pubes and the ischia, making identification of the sacrals on the actual fossil difficult. In the μCT data (Fig. 3), on the other hand, two sacral vertebrae of similar size and very short transverse process are clearly identifiable, carrying stout and distally expanded sacral ribs that articulate with the ilia. Because the sacral vertebral centra and ribs are not fused to each other, the first sacral rib on the left side has slightly shifted out of position and lies now close to the second sacral rib. The shaft of sacral rib 1 has a straight anterior margin and a slightly concave margin, whereas the opposite is seen in the slightly larger sacral rib 2. The thoracic vertebra 18 can be clearly distinguished from the two sacrals, because of its slightly curved ribs that lack lateral expansion, and which are too short to reach the ilia.

#### Caudal region

The caudal region consists of 20 vertebrae, which show a general trend in overall size and length reduction. All vertebral centra in the caudal series articulate normally with adjacent centra and none appear to be fused together. The anterior-most seven caudals (vertebrae 28 to 34) are characterised by extremely laterally projecting single headed, rod-like caudal ribs (or transverse processes), angled postero-laterally, which are not fused to the caudal centra. In the anterior-most three caudals the neural arches are again low and topped dorsally by exceedingly smaller osteoderms (reconstructed using the μCT data; similar morphology has been shown for the caudal vertebra ca11 of the holotype, Fig. 6e in ref. 5). The caudals 35 to 37 carry much shorter lateral ribs, decreasing in length, that appear fused to the centra. The lateral tip of these three caudal ribs is angled slightly towards anterior. The final ten caudals (vertebrae 38 to 47) are preserved in left lateral view and they lack transverse processes. Their overall morphology resembles that described and shown for a posterior caudal vertebra in the holotype specimen (Fig. 6f in ref. 5).

### Appendicular skeleton

#### Pectoral girdle

The pectoral girdle of PIMUZ A/III 4380 consists of the unpaired interclavicle, two clavicles, two scapulae, and two coracoids (Fig. 6). All pectoral elements are preserved in articulation, although the scapulae are slightly rotated laterally (right) and medially (left) out of their natural positions so that the glenoid portions point antero-laterally (right) and antero-medially (left) instead of ventrally. Also, because of the dorso-ventral flattening of the skeleton during fossilisation, the coracoids lost their medial articulation. The interclavicle is a flattened T-shaped element, with a single, short, and rounded anterior process and a longer tapering posterior process, which is only partly preserved where it is overlying the proximal portions of the left transverse processes of thoracic vertebra 2 and 3. The lateral projections of the T-shaped interclavicle are postero-laterally arranged, slightly wavering, and also taper into a lateral tip. Otherwise there are no striations, sculpturing patterns, or depressions visible on the ventral bone surface. As in BES SC 390, the clavicles of PIMUZ A/III 4380 are strongly angled or ‘boomerang’ shaped with ventromedial and postero-dorsal processes enclosing approximately a 70° angle. Antero-laterally, the clavicle is not expanded. The ventromedial processes taper medially and articulate in a simple overlapping fashion with the anterolateral margins of the anterior process of the interclavicle. Antero-laterally, the clavicles show a ventral shelf-like expansion, spanning the area between the two clavicular processes. Similar morphology was described in the clavicles of the placodonts *Placodus* and *Paraplacodus* ^28^. The scapula has a large crescent-shaped glenoid portion and a postero-dorsally extending, broad blade. There is no acromion process present, nor is there a clear indication for an enlarged supraglenoid process. The postero-dorsal process of the clavicle articulates medially with the scapula (Fig. 6; Supplementary Fig. S3). The left scapula has slightly rotated out of position in PIMUZ A/III 4380. The flat broad coracoids, exposed in ventral view, are roughly elliptical in shape, with only a slightly convex anterior margin and a more strongly convex posterior margin. The long axis of the bone is oriented antero-lateral to postero-medial. The thin flat bones are moulded onto covered skeletal elements (mostly onto the transverse processes of thoracic vertebral centra 3–5 and thoracic rib 3) and parts of the ventral surface are eroded so that the internal spongiosa is partially visible. There is no indication for any other surface relief such as ridge-like thickening. Antero-laterally, the coracoid margin is notched, so that the coracoid foramen would be closed off by the (non-notched) scapula margin. Together with the scapula the coracoid thus forms a ventro-laterally oriented glenoid fossa for the humerus. Medially, the left and right coracoids contact each other over a short distance (or the distal-most part of the posterior process of the interclavicle).

#### Forelimb

**Figure 7:**
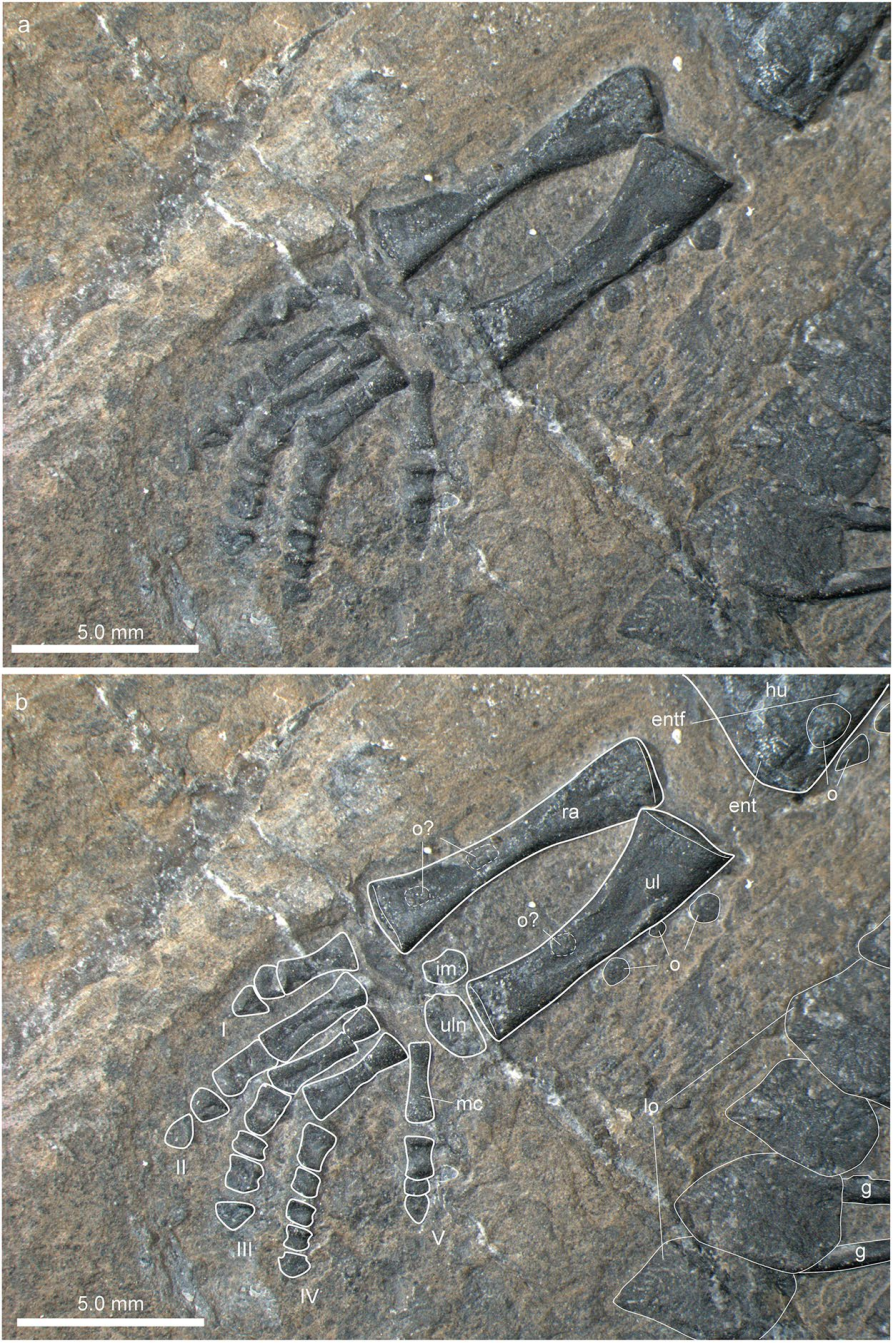
Zeugopodial and autopodial bones of the forelimb of *Eusaurosphargis dalsassoi* (PIMUZ A/III 4380). (**a**) Photograph. (**b**) Photograph overlain by interpretative drawing. Abbreviations used in the figure are entepicondyle (ent), entepicondylar foramen (entf), humerus (hu), intermedium (im), lateral osteoderm (lo), metacarpal (mc), osteoderm (o), radius (ra), ulna (ul), ulnare (uln), and digits 1 to 5 (I–V).

The configuration of the right forelimb, especially the distal aspect is more clear on the right side (Fig. 7). The humerus, which is only slightly shorter than the femur, has a straight shaft with preaxial and postaxial concave margins. The proximal and distal parts of the humerus are dorso-ventrally crushed and flattened whereas the mid-shaft region retains its ovoid shape, indicating advanced ossification in this part of the bone. The distal head is slightly more expanded than the proximal head. The proximal articular surface is convexo-concave from the preaxial to postaxial margin. The humeral epicondyles are not greatly expanded. The distal head shows an angled articulation facet anterior and posterior to the medial convexity. A weak rugose band extends only along the distal articular bone surface indicating that the synovial cartilage likely did not expand far proximally along the metaphyseal shaft. An oval entepicondylar foramen lies far proximal to the distal facet for the articulation with the ulna (contra^5^, p. 11). The presence or nature of an ectepicondylar foramen could not be recognized in the μCT scan data. The radius and ulna are of overall similar length. The preaxial and postaxial margins of the radius shaft are concave, whereas the ulna has a concave preaxial and a straight postaxial shaft margin. Both bones enclose a narrow spatium interosseum. In the radius, the proximal epiphyseal head and the distal part of the bone are of similar width. In the ulna, the proximal head is greatly expanded (the olecranon), whereas the distal portion is not expanded compared to the mid-shaft diameter. The articular surfaces of both zeugopodial elements are weakly convex.

There is only a restricted space between the zeugopodium and the autopodium, in which two carpal elements could be identified. The larger of the two, the ulnare, lies distal to the ulna, whereas the smaller one, the intermedium, lies between the distal aspects of the radius and the ulna. The ulnare is of ovoid shape with a straight proximal margin. The intermedium is kidney-shaped, with a weakly concave anterior margin.

The autopodium comprises five digits, in which the first and fifth metacarpals are shorter than the remaining ones. The first metacarpal is also stouter than the rest, and the fifth one is the most slender one of the series. The first phalangeal element is longest in digit II, followed by somewhat shorter first phalanges in digits III to V; the shortest one is found in digit I. The following intermediate phalanges are blocky or cuboid, some having a slightly incised ‘shaft’. The terminal phalanges are triangular and, especially seen in digits III and IV, wider than the penultimate phalanges. The digital formula of the hand is 2-3-4-5-3, which is the plesiomorphic condition in the pentadactyl amniote hand^29^.

#### Pelvic girdle

**Figure 8:**
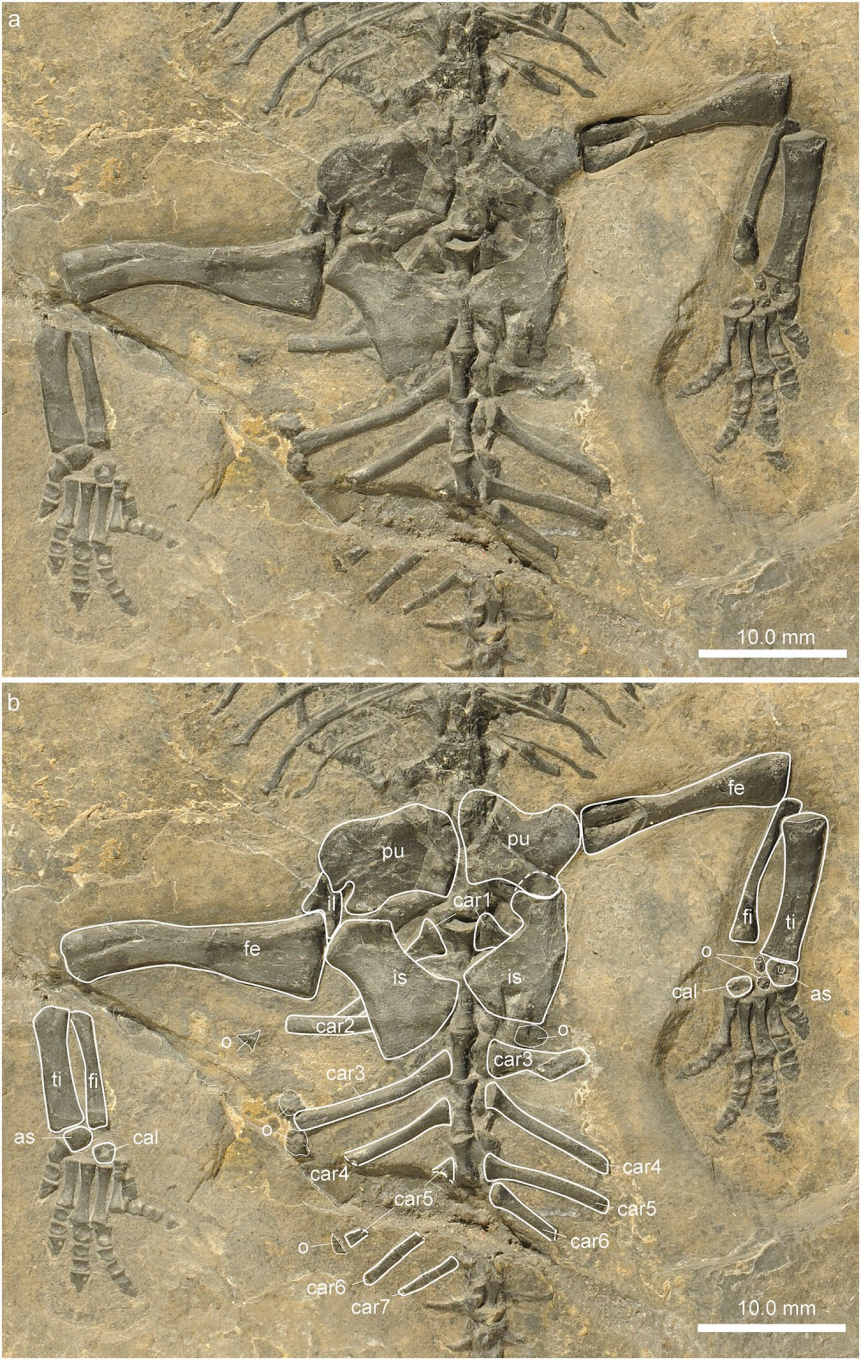
Pelvic and proximal tail region of *Eusaurosphargis dalsassoi* (PIMUZ A/III 4380). (**a**) Photograph. (**b**) Photograph overlain by interpretative drawing. Abbreviations used in the figure are astragalus (as), calcaneum (cal), caudal rib (car), femur (fe), fibula (fi), ilium (il), ischium (is), osteoderm (o), pubis (pu), and tibia (ti).

The pelvic girdle of PIMUZ A/III 4380 comprises the pubes, the ischia, as well as the two ilia (Fig. 8). The flat ventral elements, the pubes and ischia, form a large thyroid fenestra and both element pairs articulate medially. The left pubis and ischium are either affected by taphonomic deformation or a pathology and therefore deviate from the shapes of the right elements. The right pubis is of rounded rectangular shape with the long axis extends in a low angle from antero-medial to postero-lateral. The anterior and posterior margins of the right pubis are weakly and more strongly concave, respectively. The medial margin is convex. Postero-laterally, the obturator foramen forms a wide and deep notch in the pubis. The right ischium has a triangular shaped anterior articulation forming part of the glenoid. Its long axis extends from antero-lateral to postero-medial. The antero-medial and postero-lateral margins are concave forming a distinct shaft, which postero-medially continues into a fan-shaped expansion. The ilia are both mostly covered by the pubes and ischia, and the proximal head of the femur. As indicated by the μCT scan data, the glenoid part of the ilium dorsally forms into a horizontal blade, which extends posteriorly. The distal facets of the two sacral ribs articulate along the iliac blades. On the right side, the ilium has shifted slightly towards postero-lateral and thus does not contact the sacral ribs anymore.

#### Hind limb

The femur has a slightly sigmoidally curved shaft and the proximal femoral head is more expanded than the distal epiphyseal portion (Fig. 8). The left femur articulates still with the acetabulum formed by the pubis, ischium and ilium, whereas the proximal head of the right femur has shifted posteriorly so that only the anterior half of the head is still in contact with the acetabulum. The distinctly angled proximal head of the femur in ventral view indicates that this part of the bone carried a thick synovial cartilage cap during life. Distally, the condyles for the articulation with the tibia and fibula are not strongly developed. The tibia and fibula are of similar length but the tibia is much more massively built. The tibia has a straight preaxial margin and a curved postaxial one, whereas the reverse is found in the fibula. Both bones thus frame a small spatium interosseum. Both bones also show a slight expansion proximally and distally.

**Figure 9:**
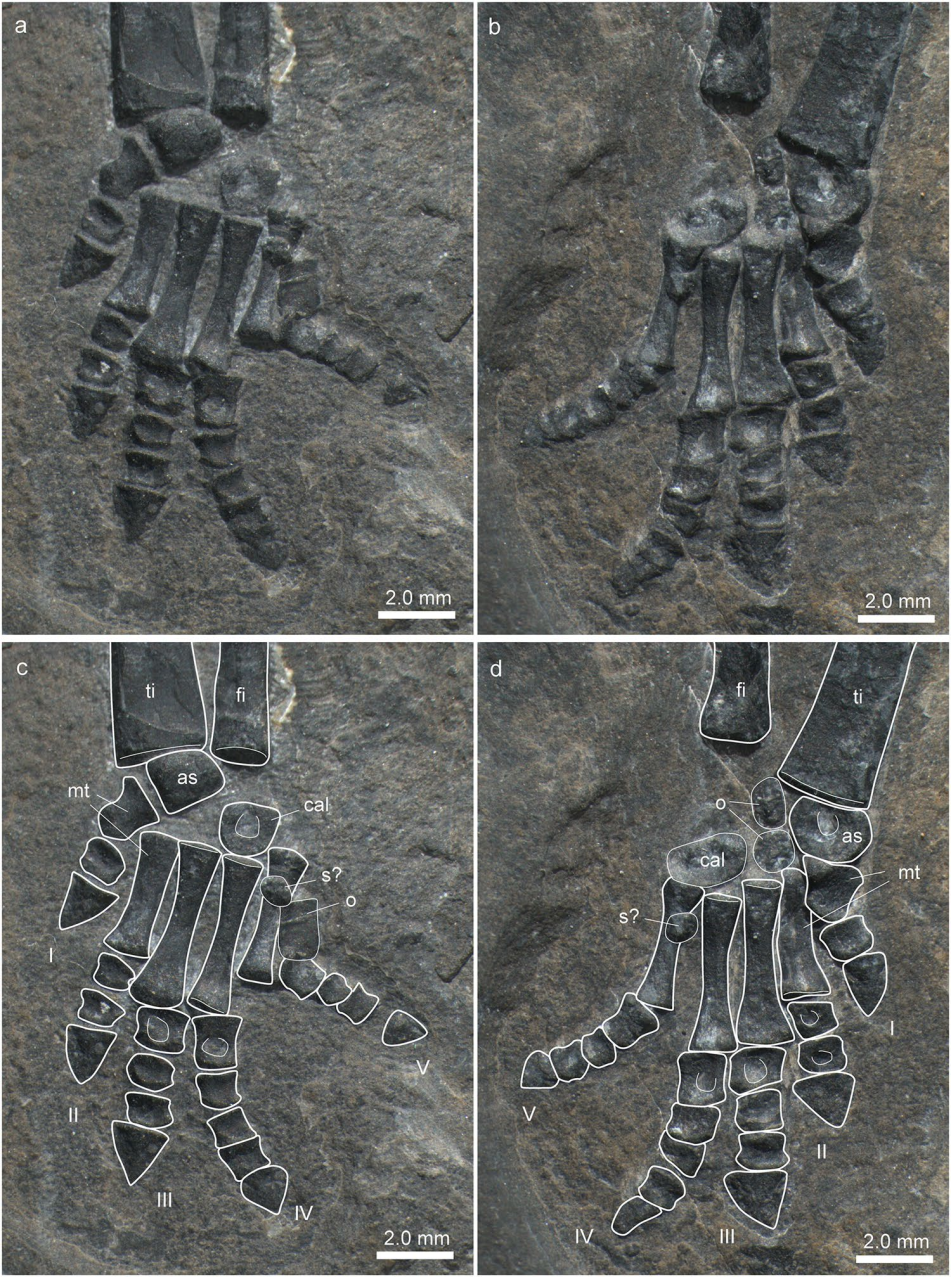
Zeugopodial and autopodial bones of the hindlimb of *Eusaurosphargis dalsassoi* (PIMUZ A/III 4380). (**a**,**b**) Photographs. (**c**,**d**) Photographs overlain by interpretative drawings. Abbreviations used in the figure are astragalus (as), calcaneum (cal), fibula (fi), osteoderm (o), sesamoid (s), tibia (ti), and digits 1 to 5 (I–V).

As seen in the left foot, the astragalus is concave proximally (Fig. 9). In the right foot the astragalus has turned so that it is not visible in strict ventral view. The calcaneum has a straight proximal border, is of ovoid shape, and lacks a calcaneal tuber posteriorly. The foot is pentadactyl, with the first metatarsal being only half the length of the other four metatarsals. The digits are short and only slightly constricted, whereas the terminal phalanges are triangular and proximally expanded in comparison to the penultimate phalanges. The digital formula of the foot is 2-3-4-5-4, which again is the plesiomorphic condition in the pentadactyl amniote foot^29^. In both feet, a small knob-like bone resting ventrally to the proximal shaft portion of the fifth metatarsal is interpreted to be a sesamoid bone preserved *in situ*. Apart from this, two osteoderms are found in the autopodium of the left and two osteoderms in the autopodium of the right foot. These elements are likely part of the axial skeleton osteoderm series (see chapter on dermal armour below). Many of the autopodial bones with an ovoid or cuboid shape (e.g., astragalus, calcaneum, and phalanges) show a central depression, indicating that the ossification here was less advanced in comparison to the periosteally deposited margins of the bones.

### Dermal armour

#### Axial osteoderms

**Figure 10:**
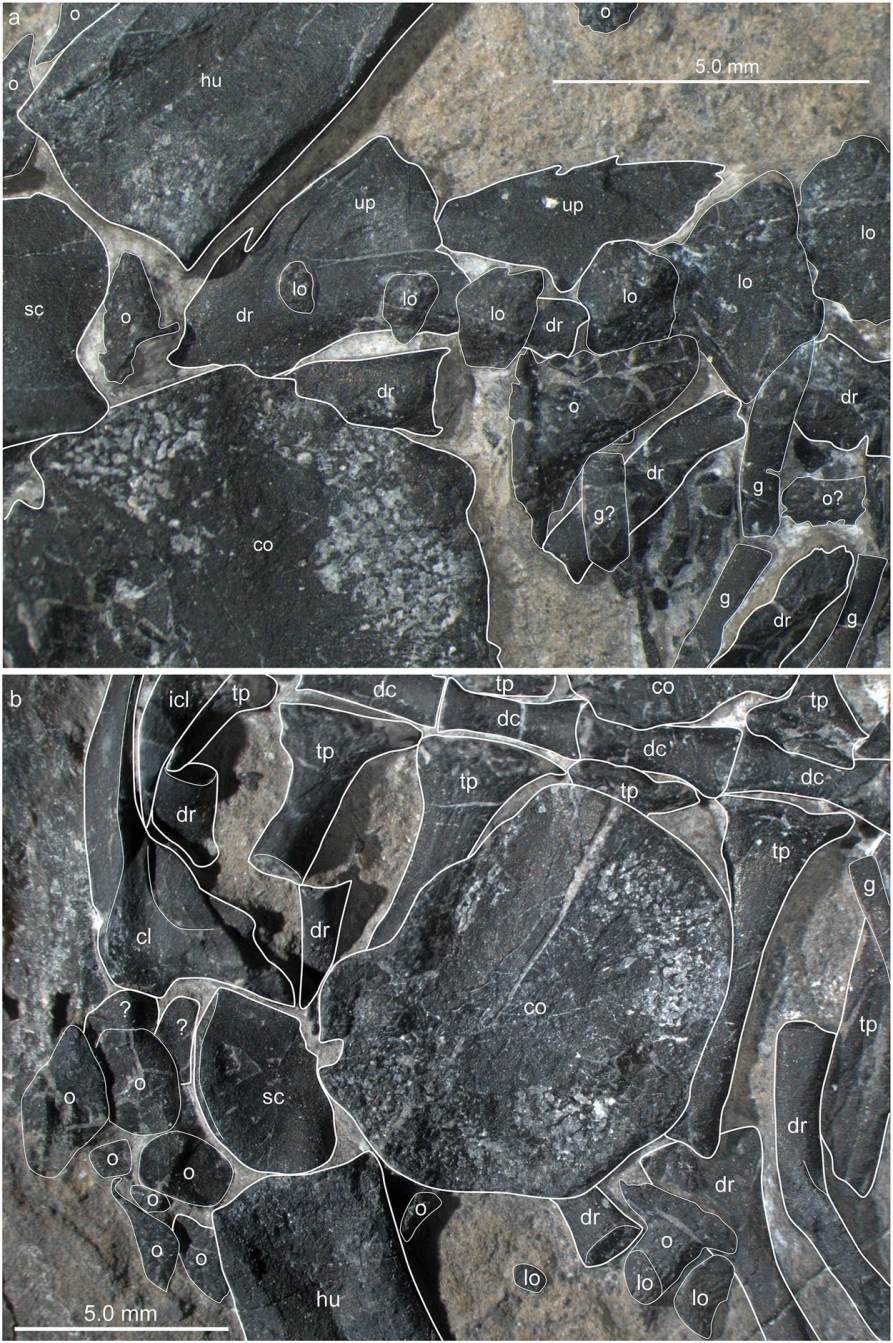
Details of the left and right lateral osteoderm series in the anterior thoracic region of *Eusaurosphargis dalsassoi* (PIMUZ A/III 4380). (**a**,**b**) Photographs overlain by interpretative drawings. Abbreviations used in the figure are clavicle (cl), coracoid (co), thoracic/dorsal vertebral centrum (dc), thoracic/dorsal rib (dr), gastralia (g), humerus (hu), interclavicle (icl), lateral osteoderm (lo), osteoderm (o), uncinate process (up), scapula (sc), and transverse process of thoracic/dorsal vertebra (tp).

PIMUZ A/III 4380 shows a number of dermal ossifications that are closely associated with the underlying endoskeleton. As revealed by the μCT scan data (Fig. 3), a medial row of low, conical osteoderms with roughly triangular bases was found directly dorsal to each neural spine extending from the 3^rd^ cervical vertebra to the anterior caudal region (28 in total). In the cervical and mid-trunk regions, a few osteoderms have slightly shifted off the neural spine, indicating that there was no fusion but loose attachment between dermal armour and the underlying skeletal elements. In more mature specimens, as in the holotype BES SC 390^5^, there is indication that osteoderms can be fused to the underlying endoskeleton, i.e., the neural spines (e.g. cervical c2 in the holotype). As the last osteoderms are increasingly reduced in size, the posterior portion of the tail was likely osteoderm-free. Secondly, again mostly visible in the μCT scan, there are also a single left and right row of large, conical osteoderms with broad ovoid bases which extend from the shoulder girdle (at the height of the 3^rd^ dorsal rib) backwards to the last dorsal rib. All conical osteoderms (13 on each side) show a pointed, slightly off-centred apex and an uneven broadened base, which is ventrally flat to slightly concave (Fig. 10). The external bone surface shows tiny pits and few longer grooves at the base margins. It is thus likely that the spiked osteoderms were closely related to the uncinate processes of the dorsal ribs or were at least positioned slightly adjacent to them. A similar condition was described for a dorsal rib of aff. *Eusaurosphargis* from Winterswijk, which carries a triangular and rugose uncinate process (NMNHL Wijk10-246^7^, National Museum of Natural History (NCB Naturalis), Leiden, The Netherlands) and in the holotype BES SC 390 (e.g., dr_6_ in Fig. 14 in ref. 5).

**Figure 11:**
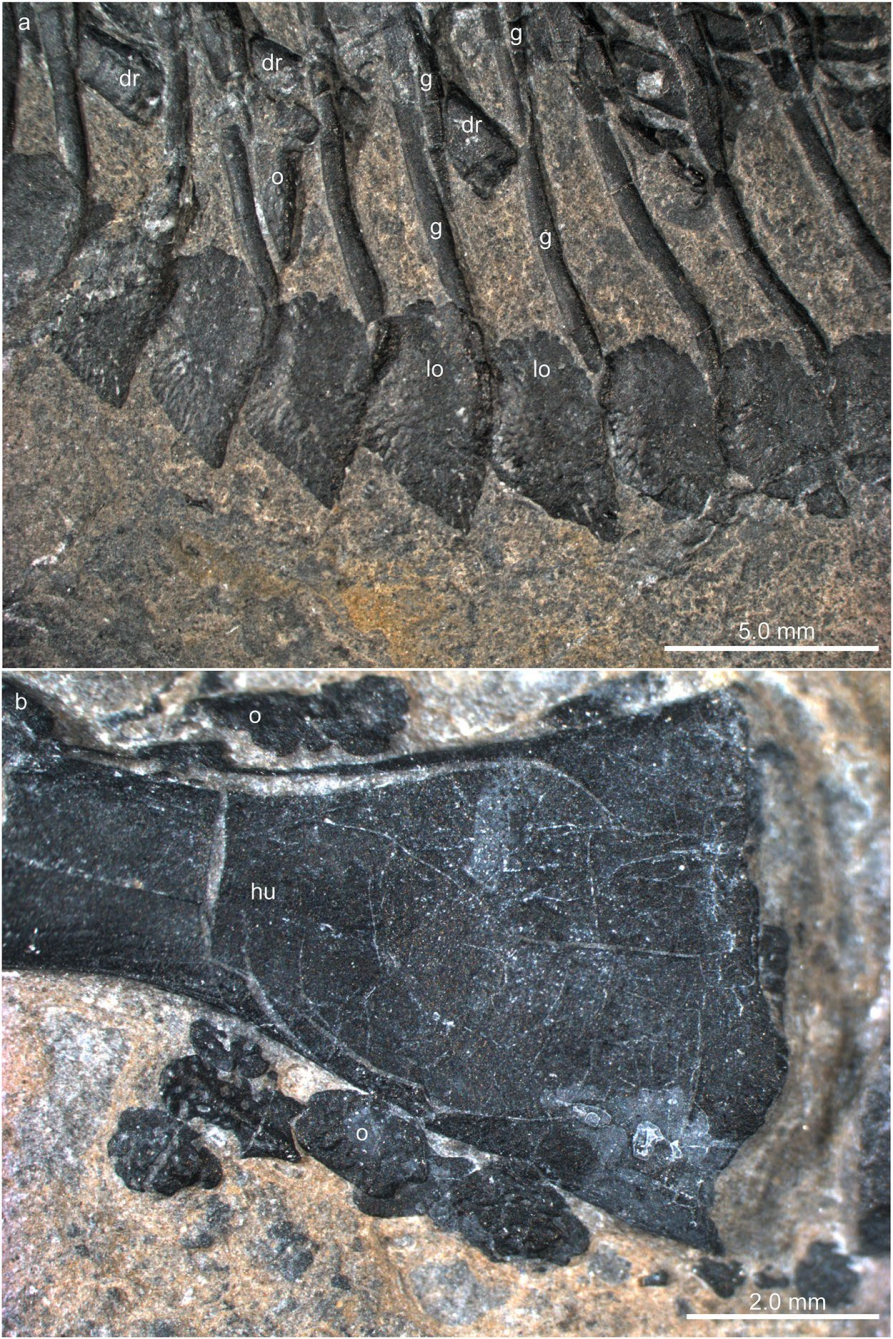
Detail of the dermal armour of *Eusaurosphargis dalsassoi* (PIMUZ A/III 4380). (**a**) Lateral trunk osteoderms and associated gastralia. (**b**) Distal part of the left humerus with associated pre- and postaxial osteoderms. Note sculpturing pattern and keel of some of the osteoderms. Abbreviations used in the figure are thoracic/dorsal rib (dr), gastralia (g), humerus (hu), lateral osteoderm (lo), and osteoderm (o).

Laterally the trunk region of PIMUZ A/III 4380 is covered by a single row of shingle-like osteoderms that partially overlap with each other in that the anterior margin of one osteoderm partially covers the posterior margin of the preceding osteoderm (Fig. 11a). On each side, there are 28 osteoderms in total. Starting with the 5^th^ osteoderm from anterior (Fig. 10), each osteoderm is resting between two gastralia (Fig. 11a). The anterior-most (nos 1–3) and posterior-most (nos 22–28) osteoderms are smaller, more roundish and lacking a clear long-axis, whereas the remainder within the series (nos 4–21) are asymmetrical in shape, with an anterior convex margin, a straighter posterior margin, and a tapering lateral point. Each osteoderm shows a rugose external surface with slightly elongated and branching troughs directly at the lateral margins (indicating that this part was likely covered by a keratinous spiky scute as is found in modern reptiles). Proximally, the posterior portions of the osteoderms do not show rugose surfaces (Fig. 11a).

There are four osteoderms on the right side lateral to the longest caudal ribs in the proximal tail region and three on the left side. Although their shape is difficult to discern on the fossil, all show an off-centred apex over an irregular base in the μCT scan, probably constituting an extension of the two lateral rows of spiky osteoderms of the trunk region into the proximal tail region.

On the left side of the specimen, several rectangular shaped and transversely oriented structures were found that represent flat, platy osteoderms overlying the caudal ribs dorsally. No comparable structures were visible in the μCT scan on the right side of the body.

#### Appendicular osteoderms

Osteoderms are found both together with pectoral girdle elements and the forelimbs, whereas an association with the pelvic girdle and the hind limbs is less clear (with the exception of the two shifted caudal osteoderms mentioned above). Large triangular, partially overlapping shingle-like osteoderms are covering the lateral part of the clavicle, the anterolateral margin of the scapula, as well as the complete preaxial margin of the humerus, creating an anterior serrated ridge of overlapping osteoderms (nine on the left side and five on the right side). Anterior to the scapula, three more flat, round osteoderms (internal bone surface is exposed), which are partially overlapping each other, were recognised in addition to the shingle-like osteoderms. Postaxially, up to 11 osteoderms are found adjacent to the distal region of the humeral shaft (Fig. 11b) and along the ulna, the largest of which also show a ridged morphology. The smaller osteoderms have a more roundish shape and less pronounced pits on the external bone surface.

Besides the continuation of the lateral osteoderms up to the caudal region as mentioned above, the sacral/pelvic region and the hind limb also show a few osteoderms. The right region of the pelvis is covered dorsally by five thin rectangular plates of uneven size, four of which are arranged in a medio-lateral fashion. These platy osteoderms, which were only found on the right side in the μCT scan might have reinforced the space between the dorsal and lateral spiked osteoderms in the pelvic region. No osteoderms were found associated with the stylopodium and the zeugopodium of the hind limb, whereas several osteoderms were encountered in the autopodial region. In both feet, a small round bone lies ventral to the proximal shaft region of the metatarsals 5, exactly in the same spot (Fig. 9c,d). In the right foot, an additional thin flat rectangular bone, reminiscent of those covering the pelvic region dorsally, lies directly adjacent postaxially to metatarsal 5. In contrast, two small round and spiky osteoderms were found between the tarsal bones, the astragalus and the calcaneum, of the left foot. As the rectangular osteoderms are only preserved on the right side and the two small osteoderms resting between the tarsal bones were only found on the left side in PIMUZ A/III 4380, we hypothesize that these “drifted” to their respective positions post-mortem, whereas the small bones ventral to the metatarsals5 represent sesamoid bones preserved *in-situ*.

In this case, the two small spiky osteoderms discovered among the tarsal elements are interpreted to belong to the caudal osteoderm series, in which case these elements would have shifted about 10 mm from their original position lateral to the caudal ribs.

#### Gastralia

The gastral basket (Fig. 2b) covers the thoracic vertebral series from thoracic centrum 5 to 18, starting at the posterior margin of the coracoids and extending almost to the sacral region. The gastral basket consists of 24 gastral units, with each gastral unit being composed of five individual elements: a medial one with a thickened and slightly angled median part and simple tapering lateral prongs, and two lateral rod-like elements on each side, of which the lateral-most element is almost twice as long as the intermediate element. Each of the gastral elements taper laterally and the elements overlap to a large degree, often by more than 50 percent of their respective length. The lateral-most tapering tips are slightly bent anterodorsally, between which a single shingle-like osteoderm is present (see above).

### Phylogenetic analyses

**Figure 12:**
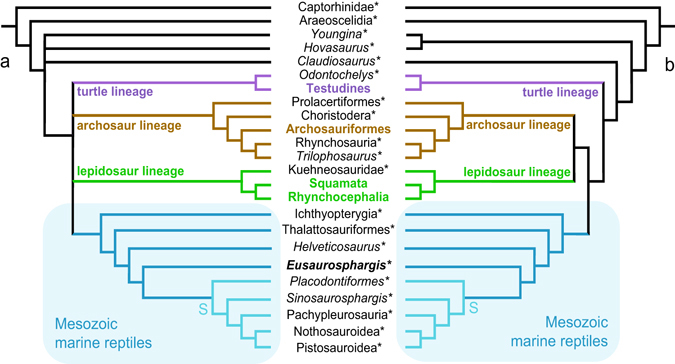
Phylogenetic analyses of diapsid reptilian lineages, showing the position of *Eusaurosphargis dalsassoi* among the Mesozoic marine reptiles. The three crown-group lineages of modern reptiles and the Mesozoic marine reptilian lineages are marked in colours; extinct taxa are marked by a small asterisk. (**a**) Analysis 1, run with the updated scoring for *E*. *dalsassoi* in the first matrix^30^. (**b**) Analysis 8, run with the updated scoring for *E*. *dalsassoi* in the third matrix^30^, with ‘all zero ancestor’ (not shown) as outgroup. Note that once the ‘all zero ancestor’ is excluded from the analysis and Captorhinidae is chosen as an outgroup, the tree topology is congruent with that of (**a**). Sauropterygia (S).

First, the scoring of *Eusaurosphargis dalsassoi* was updated (see Supplementary Note; Supplementary Figs S4–S11) based on the new specimen PIMUZ A/III 4380 and implemented in the 140 character/44 taxa matrix (first matrix^30^). Compared to the original dataset, 81 characters (57.9%) out of 140 could now be scored (24 more characters, 17.1%). This Analysis 1 recovered 16 most parsimonious trees (MPTs) of 574 steps, the strict consensus of which (Fig. 12) shows a polytomy of the modern reptile clades (turtle, lepidosaur, and archosaur lineages) with a monophyletic clade of the extinct marine groups (Ichthyopterygia (Thalattosauriformes (*Helveticosaurus* (*Eusaurosphargis* (Sauropterygia)))))). Furthermore *Sinosaurosphargis* was found to be the sister taxon to Eosauropterygia, and *Hanosaurus* and *Wumengosaurus* were lying on the pachypleurosaur lineage.

In a second step, the analysis was re-run with characters 90, 113, 114, 120, 126, 127, and 131 deleted (corresponding to characters 50, 62, 63, 67, 71, 74, and 120 of the list of potentially problematic ‘marine reptile’ characters listed in the appendix of ref. 31; see discussion below). This Analysis 2 recovered three most parsimonious trees of 537 steps, with the strict consensus showing better resolution of the modern reptile clades, but far less resolution among the Mesozoic taxa.

In a third analysis the *Eusaurosphargis dalsassoi* scoring was added to the 213 character/41 taxa matrix (the second matrix^31^). The taxon could be scored for 107 (50.2%) out of the 213 characters. The strict consensus of 12 MPTs with 845 steps shows overall good resolution within the archosaur and lepidosaur clades, but less resolution within the marine lineages: (Thalattosauriformes (*Wumengosaurus* (Ichthyopterygia, Hupehsuchia) (Saurosphargidae (*Eusaurosphargis*, *Helveticosaurus*, *Placodus*, Eosauropterygia)))). In this Analysis 3 the position of *Eusaurosphargis* and *Helveticosaurus* in relation to the sauropterygians could not be resolved.

In a fourth step, the same analysis was re-run with the problematic ‘marine reptile’ characters 50, 62, 63, 67, 71, 74, 114, 120, 151, 171 (10 out of the whole set of ‘problematic’ characters listed in the appendix of ref. 31; see discussion below) deleted. The Analysis 4 yielded 34 MPTs with 810 steps, the strict consensus of which is overall poorly resolved. The extant archosaur and lepidosaur lineages are found in a polytomy with Choristodera (usually recovered on the archosaur lineage as well), and a (Thalattosauriformes (*Wumengosaurus* (Ichthyopterygia, Hupehsuchia) (Saurosphargidae (*Eusaurosphargis*, *Helveticosaurus*, *Placodus* (Eusauropterygia))) clade.

The overall resolution of the cladogram was even lower still (fifth step), when the whole set of 18 ‘problematic’ characters was excluded (Analysis 5, resulting in 46 MPTs with 787 steps), in which most of the non-thalattosaurian marine taxa and subclades collapsed into a large polytomy together with the clade comprising Hupehsuchia and Ichthyopterygia.

In the sixth step, deleting *Eusaurosphargis dalsassoi* improved the topology only slightly (Analysis 6, strict consensus of 28 MPTs with 768 steps) by recovering resolution within Sauropterygia again (*Helveticosaurus* and *Placodus* form a sister group, which is then sister to Eosauropterygia).

In a seventh and eighth step, the updated scoring of *Eusaurosphargis dalsassoi* was implemented in a third matrix^13^, which includes a much larger sample of placodonts compared to the matrix used by^30^, and a slightly changed character, no. 138, to better differentiate the variously armoured placodonts). Running this Analysis 7 with an ‘all-zero ancestor’ as outgroup (resulting in 4 MPTs with 614 steps) led to overall slightly better resolution compared to Analysis 1, and *E*. *dalsassoi* as sister taxon to Sauropterygia (including *Sinosaurosphargis*). On the other hand, excluding the ‘all-zero ancestor’ and using Captorhinidae as outgroup (Analysis 8; resulting in 16 MPTs with 612 steps) led to the same tree topology as found in Analysis 1 (except different placodont ingroup relationships due to the increased amount of placodont taxa). Compared to the original tree topology presented by ref. 13: Fig. 7), Ichthyopterygia are again sister to all remaining Mesozoic marine taxa and *Sinosaurosphargis* was recovered as sister taxon to Eosauropterygia instead of being sister taxon to the whole Sauropterygia clade.

## Discussion

With the description of this new specimen of *Eusaurosphargis dalsassoi* we confirm that the completely disarticulated holotype BES SC 390 indeed represents a single individual. Most elements previously described^5^ could be identified in PIMUZ A/III 4380 and, in most cases, the morphology of the new specimen closely matches the description of the holotype, although there are a few exceptions (see Supplementary Fig. S12). The preservation of PIMUZ A/III 4380 on the bottom side of the limestone slab indicates that the specimen was embedded with its ventral side resting in the soft sediment, while the dorsal side was exposed to the water column. Consequently, the ventral bones are found *in situ* (up to most of the tiny osteoderms), whereas some of the dorsal elements (scapulae, some ribs and larger dorsal osteoderms and the skull and lower jaw remains) shifted to some extent from their original position in the skeleton. In the case of the head, most of the palatal region is dislocated but still associated with each other, although the whole skull portion was displaced posteriorly to lie adjacent to the shoulder girdle on the right side. The facial bones and those comprising the skull roof then were also displaced to the left and right side of the vertebral column.

The new data gained from PIMUZ A/III 4380 complement in part those based on the holotype BES SC 390, especially in the palatal region, which was previously poorly understood. PIMUZ A/III 4380 shares with *Palatodonta bleekeri* ^14^ (and placodonts such as *Paraplacodus broilii* and *Placodus gigas*) the deep skull shape and wide snout with large external nares, as well as the double tooth row in the upper jaw (on the maxillae and palatines) and a single row in the lower jaw. On the other hand, the lower jaw of *E*. *dalsassoi* was much more robustly built and the homodont teeth of the premaxilla, maxilla and palatine bone contrasts with the dentition of *P*. *bleekeri*, which includes an array of pointy teeth in the maxilla and palatine and chisel-like teeth in the premaxilla^14^.

By virtually reconstructing the thoracic ribs it became apparent that they all carried uncinate processes of different shapes. In the first three thoracic ribs the uncinate processes are also pointing anteriorly, whereas in the following ribs they are pointing posteriorly (thus character 195 in the second matrix^31^ was scored polymorphic ‘[1,2]’ in *Eusaurosphargis dalsassoi*). Comparison with BES SC 390 also indicates that the elements identified in the holotype as ‘car1-car3’^5^ (p. 9, Fig. 15) are not the purported anterior-most caudal ribs, but indeed resemble the sacral ribs of PIMUZ A/III 4380 in shape (‘car1’: sacral rib 1; ‘car2’ and ‘car3’: left and right sacral rib2). Accordingly, the simpler, elongated rod-like elements ‘car4’, ‘car6-car9’, and ‘car12’ represent the six anterior-most caudal ribs in PIMUZ A/III 4380.

Both girdles are mostly preserved *in situ* in PIMUZ A/III 4380. The clavicles appear to abut each other medio-dorsally in front of the interclavicle as was described for example in the sauropterygian *Corosaurus alcovensis* ^32^ or the younginiform *Hovasaurus boulei* ^33^, whereas an interdigitation of both clavicles is not present. In contrast to *Corosaurus*, the posterior process of the T-shaped interclavicle is well developed and not reduced, resembling the interclavicle of the Permian archosauromorph *Protorosaurus speneri* ^34^ or the Middle Triassic tanystropheids *Tanystropheus* and *Macrocnemus* ^35^; T.M.S. personal observation). Because the coracoids have shifted only little if at all in PIMUZ A/III 4380, they did not articulate to a large degree medially with the interclavicle, but were rather articulating with the tip of the interclavicular posterior process or, more likely, with each other medially just posterior to the interclavicle (framing a large space). It could be speculated that, as in modern squamates, *Sphenodon punctatus*, and crocodylians^24^, the pectoral girdle bones were embedded in cartilage or in resilient connective tissue, which is not preserved in the fossil, but which kept the girdle bones in place during decomposition of soft parts and initial diagenesis.

As noted above, the flat ventral elements of the pelvic girdle, the pubes and the ischia, frame a large thyroid fenestra. This contrasts with the original reconstruction of the pelvic girdle based on the holotype BES SC 390. The ischium of the holotype was reconstructed and described to be kidney-shaped, with a convex anterior margin (the posterior margin is not completely preserved) and an upwards-facing acetabular portion^5^. It was thus described as being similar in outline to the ischium of *Helveticosaurus zollingeri* (PIMUZ T 4352^9, 10^) from the Anisian-Ladinian boundary (Besano Formation) of Monte San Giorgio, southern Switzerland. The ischia of *Helveticosaurus* are only fragmentarily preserved and much of the outline of the bones has been restored^10^. A kidney-shaped plate-like ischium with an anterior convex margin has been described also in the placodont *Pararcus diepenbroeki* from the early Anisian (Vossenfeld Formation; lower Muschelkalk) of Winterswijk, The Netherlands^30^ and *Largocephalosaurus qianensis* from the Anisian (Guanling Formation) of Guizhou Province, southwestern China^11^. The right ischium of PIMUZ A/III 4380, however, differs starkly from the presumed kidney-shaped ischia of BES SC 390 (and of those of *Helveticosaurus*, *Largocephalosaurus*, and *Pararcus*) in having anterior and posterior concave margins (well preserved and visible only in the right ischium), forming a flat, slightly fan-shaped element with a slightly asymmetrical distally expanded portion. The ischium shape is thus more reminiscent of the general shape of the ischia of the Late Triassic sauropterygian *Bobosaurus forojuliensis* ^36^ or the Early Triassic hupehsuchian *Hupehsuchus nanchangensis* ^37^, although the distal expansion is much more pronounced in these taxa.

Based on this comparison we re-identify the only partially preserved bone originally identified as the ischium in holotype BES SC 390 as a badly preserved pubis in which the marginally open obturator foramen is not well visible. On the other hand, the two bones originally identified as the pubes (p1 and p2 in ref. 5 match more the shape of the well preserved right ischium of PIMUZ A/III 4380, thus representing the two ischia of the holotype specimen accordingly.

There is a slight asymmetry between the left and right side of PIMUZ A/III 4380, which is especially pronounced in the pelvic region. This is evidenced by a deformed pubis and ischium, the proximal part of the left femur, shortened caudal ribs 1–3, the last of which carrying a deformed articular head and a broken and displaced distal tip on the left side. The left caudal ribs 1–3 appear shorter compared to the equivalent bones on the right side. On the other hand the sacral ribs, the ilium and the femur appear to be unaffected.

There are two possible explanations for the slight distortion of the otherwise exquisitely preserved specimen. Explanation 1 could be that a trauma or infection causing the malformation of the bones in the pelvic region occurred early during the life of the animal, thus leading to a pathologic condition. Explanation 2 could be that some bones of the pelvic region did not sink as deeply into the soft sediments and were thus not embedded in a perfect horizontal position (as evidenced by the angled embedding of the left humerus). This could have caused a taphonomic distortion of individual bones during compaction and lithification of the sediment.

The new specimen, which is about 1/3 smaller than the BES SC 390 and thus likely a young individual, also allows us to reinvestigate and clarify the anatomy of some skeletal elements of the larger, slightly more mature holotype specimen. The still early ontogenetic stage of PIMUZ A/III 4380 is corroborated by the disarticulation of the skull elements in an otherwise fully articulated specimen, the presence of only two carpal and tarsal elements in the hand and foot, as well as in the separation of many transverse processes from the vertebrae.

The high level of articulation of the skeleton of PIMUZ A/III 4380 enabled us to reconstruct the complex dermal armour of *Eusaurosphargis dalsassoi* in detail. Even in a juvenile, the armour was extensive, with osteoderms covering the shoulder region, the forelimbs, the flanks of the trunk both dorsally and ventrally, and the pelvic region and proximal part of the tail. Among the Mesozoic marine reptile groups (Fig. 12), hupehsuchians, placodonts (except *Paraplacodus broilii*), saurosphargids, and *Eusaurosphargis dalsassoi* show extensive dermal armour, whereas none of the members of ichthyopterygians, thalattosaurs and eosauropterygians carry any osteoderms. *E*. *dalsassoi* shares with the placodont genus *Placodus* a simple row of osteoderms above the vertebral column, although the shape and positioning of the osteoderms is different in both taxa^38, 39^. The row of overlapping lateral osteoderms that are closely associated with the gastral apparatus and the dorsolaterally pointing spiky osteoderms on the trunk are unique features of *E*. *dalsassoi*.

*Eusaurosphargis dalsassoi* is known only from three more or less complete specimens (the holotype BES SC 390 from Besano, the new referred specimen PIMUZ A/III 4380 from Ducanfurgga, and a yet undescribed disarticulated skeleton as part of slab NMNHL RGM 449487 from the Netherlands^27^ and a few isolated finds; which makes the taxon one of the rarest faunal components in the three Middle Triassic localities). Given the large number of pachypleurosaurs of similar size range, among a plethora of thousands of other fossils, we corroborate the previous idea that *E*. *dalsassoi* had a terrestrial habitat preference. Besides the scarcity of *E*. *dalsassoi*, there are also anatomical features that support a terrestrial lifestyle. Although the proportions of the hand, ulna and humerus and number of carpal and tarsal elements of PIMUZ A/III 4380 are similar to those of small pachypleurosaurs from the Besano and Meride formations of Monte San Giorgio (such as *Neusticosaurus pusillus* or *Serpianosaurus mirigiolensis* ^40, 41^; T.M.S. personal observation), especially the autopodial elements are much more robust, including wide spade-like terminal phalanges instead of tapering ones. PIMUZ A/III 4380 also has angled and defined articulation facets in the distal epiphyseal regions of the humerus when compared to the pachypleurosaurs, making a stiffening of the elbow and region less likely in *E*. *dalsassoi*. In addition, the hind foot is also short and robust, again sporting spade-like terminal phalanges (reminiscent of those of fossorial animals^42^), whereas the short and proximally dorso-ventrally wide tail would be similarly inefficient in providing propulsion. Lastly, the stylopodial elements of PIMUZ A/III 4380 (seen in the μCT scan data; Supplementary Fig. S13) are tubular, moderately thin-walled bones with large marrow cavities. Such an internal bone microanatomy is usually found in terrestrial and amphibious, but not in marine, diapsids^43, 44^. Based on these different lines of evidence, we conclude that an essentially terrestrial lifestyle is most plausible for *E*. *dalsassoi*, although possible excursions into the aquatic medium cannot be ruled out. As such it resembles yet another allochthonous element among the otherwise marine fauna recorded in the Prosanto Formation.

Recently it was argued that several phylogenetic characters, which are explicitly linked to an aquatic lifestyle, bias the results of phylogenetic analyses, thus favouring an all-aquatic ‘super-clade’ that includes hupehsuchians and ichthyopterygians, sauropterygians, saurosphargids, and thalattosauriforms^31^. Re-coding these phylogenetic characters in question as unknown (as ‘?’) – a so-called ‘un-coding’ – only among the marine reptiles led to a split-up of the groups into a clade (Thalattosauriformes (*Wumengosaurus* (Hupehsuchia, Ichthyopterygia))) situated among basal diapsids, and a more highly nested clade ((*Helveticosaurus*, Lepidosauria) (Saurosphargidae, Sauropterygia)), as sister to the archosaur lineage.

Although we agree with the concept that some characters functionally related to a marine lifestyle might influence phylogenetic analyses, we also see several problematic aspects. Firstly, we find the ‘un-coding’ of characters to question marks in only a subgroup (the Mesozoic marine reptiles) inconsistent. Instead we argue to either keep or to delete those highly ‘problematic’ characters completely from the analyses. Secondly, we argue that not all eighteen proposed characters (characters 1, 9, 50, 58, 61, 62, 63, 67, 68, 71, 74, 114, 120, 145, 151, 171, 180, and 186 of ref. 31) can be unambiguously linked to a marine lifestyle, partly because some characters rather reflect special feeding specialisations and changes of skull bones and musculature linked with them (e.g., characters 1, 9, and 180), and partly because other characters yield strongly inhomogeneous results for clearly marine forms (character 58, 61, 68, 145, and 186). Thirdly, the lifestyle of some taxa might have been misidentified a priori and thus would be influenced by the partial ‘un-scoring’. One example is *Claudiosaurus germaini* Carroll, 1981, which is indicated to be a terrestrial taxon in ref. 31 (Fig. 7), and later in the appendix noted as a potentially amphibious taxon (in accordance with the original description^43^). In contrast, in more recent studies using bone histological data, *C*. *germaini* is indicated to be a secondarily aquatic taxon, which inhabited shallow marine environments^44–47^.

Furthermore, as shown by Analysis 2, deleting even a small number of the ‘problematic’ characters has greater impact on the topology recovered from a dataset which focuses on Mesozoic marine, especially sauropterygian, forms (27 marine taxa in analysis 1 and 2), whereas the impact is less severe in the dataset which includes far fewer Mesozoic marine forms (15 marine taxa in analyses 3 to 6). In general, deleting a subset or the full set of ‘problematic’ characters from the analyses generally led to a loss of resolution by introducing unresolved polytomies at different areas of the cladograms in comparison to analyses 1 and 3.

According to our Analysis 1 and Analyses 7 and 8, *Helveticosaurus zollingeri* and *Eusaurosphargis dalsassoi* represent successive outgroup taxa of Sauropterygia (which potentially includes the clade Saurosphargidae as well, because of the inclusion of *Sinosaurosphargis*). In those analyses, the node including Ichthyopterygia as sister to the remaining Mesozoic marine taxa was supported by a number of homoplastic traits but no unambiguous synapomorphies (unequivocal characters 7, 52, 112, 113*, 114*, and 126*; potentially ‘problematic’ characters sensu^31^ marked with an asterix) and the node has a moderate Bremer support of 3. The node grouping *Eusaurosphargis dalsassoi* and Sauropterygia had only a low Bremer support of 1 but the node was supported by four shared characters, the latter three of which could now be addressed through the anatomy of the new specimen (unequivocal character 98(1): clavicle applied to medial surface of scapula; 108(1): pectoral fenestration present; 123(1): thyroid fenestra present). Of these characters, character 108 represents an unequivocal and unambiguous synapomorphy.

Analysis 3, in comparison, recovered *H*. *zollingeri* and *E*. *dalsassoi* in a polytomy with *Placodus* and Eosauropterygia. In this analysis the node grouping thalattosaurs with the remaining Mesozoic reptile groups was supported by nine synapomorphies (unequivocal characters 50*, 62*, 63*, 99, 114*, 140, 151*, 180*, 186*; again potentially ‘problematic’ characters are marked with an asterix) and a Bremer support of 1, whereas the clade *Eusaurosphargis*, *Helveticosaurus*, and the sauropterygian taxa is weakly supported only by a single unequivocal shared trait (character 89) and also a low Bremer support of 1.

In conclusion, as exemplified by the different analyses run herein, a topology as shown by the preferred tree of ref. 31 (Fig. 7b; which is reminiscent of the results of the analysis of ref. 48: Fig. 2, except that thalattosaurs and ichthyoptygians + hupehsuchians do not form a sister clade), could not be recovered. Instead, the results of the Analyses 1 and 2 (based on the first matrix^30^) and Analyses 3–6 (based on the second matrix^31^), consistently recovered a monophyletic clade of Mesozoic marine taxa with various levels of support, but always including thalattosaurs, ichthyopterygians, hupehsuchians, saurosphargids, sauropterygians, *Helveticosaurus zollingeri* and *Eusaurosphargis dalsassoi*. Furthermore, in the better supported Analyses 1 and 8 (see Fig. 12), our preferred analyses herein, *Eusaurosphargis dalsassoi* represents the sister taxon to Sauropterygia (including *Sinosaurosphargis*). The inferred terrestrial lifestyle of *E*. *dalsassoi* (see chapter on Palaeoecology above) would thus represent a reversal from aquatic habitats rather than retention of an ancestral terrestrial condition.

## Methods

PIMUZ A/III 4380 is from the Prosanto Formation and comprises a complete skeleton, prepared in ventral view on the underside of a thin limestone slab (Fig. 2). The specimen was discovered in 2002 by C.O. during a bed-by-bed excavation near the locality Ducanfurgga in a fossiliferous level of the Upper Prosanto Formation (bed no. 1, Early Ladinian, Middle Triassic), directed by H.F. The broken unknown specimen was identified as a thin brownish-black streak only within the sediment matrix. It was collected piece by piece and then re-assembled, before mechanical preparation was carefully performed in 2014–2015 by C.O.

Given its small size and the fragility of the articulated bones, the specimen could not be prepared completely out of the matrix. To address the dorsal aspects of its morphology (Figs 3 and 4), micro-computed tomography (μCT) was conducted at Empa, Dübendorf, Switzerland, with a μDETECT setup. In this instrument, X-rays are generated by a Finetec micro-focus X-ray tube (model FOMR 300.03Y RT) operating at an acceleration voltage of 300 kV and a tube current of 170 µA. A 1 mm copper filtration plate was used to harden the beam. Projection images were acquired by a Perkin Elmer flat panel detector (model XRD 1611-CP3) used in its 2 × 2 binning mode, corresponding to pixels of 200 μm. An acquisition time of 1.75 s was employed and a total of four frames were averaged per orientation of the sample. The sample was rotated in 1600 steps around a fixed axis in increments of 0.225°. The radiographs were corrected with adequate image processing techniques in order to avoid ring artefacts in the reconstruction. The images were reconstructed afterwards by employing an Empa in-house implementation of the Feldkamp-Davis-Kress algorithm^49^. The resulting voxel size of the 3D volume is 84 μm. It was only by using the μCT scan data that the morphology of many of the skull bones, the thoracic ribs, the dorsal trunk osteoderm cover, as well as much of the vertebral column (e.g., vertebral centra; osteoderms dorsally topping the low neural arches) could be reconstructed.

To assess the systematic position of the new fossil, it was coded into two diapsid matrices (the first^30^ and third matrices^13^, which are based on the analysis of ref. 14 and references therein, with Captorhinidae as an outgroup), as well as an amniote matrix (the second matrix^31^, which is mostly based on the matrix of ref. 50, with Seymouriidae as outgroup) with additions from ref. 11, as well as the inclusion of some new characters. All phylogenetic analyses were run using the Windows version 1.5 of TNT^51^. The ‘Traditional Search’ of TNT was used as search option, and each was run with 30000 trees in memory, 10000 replicates, and 100 trees saved per repetition, as well as the Tree Bisection Reconnection (TBR) mode activated. In addition, all characters were equally weighted and treated as unordered. The resulting cladograms were then modified with Windows version 3.10 of Mesquite^52^ and Adobe Creative Suite.

Where possible, measurements were taken directly on PIMUZ A/III 4380 using iGAGING OriginCal digital callipers. In case of minute bony elements such as the hyoids, measurements were also taken on digital images using ImageJ 1.47k^53^.

Given that numerous osteoderms occupy different positions on the body of PIMUZ A/III 4380 and that their orientation might not always be clear, the osteoderm side facing away from the body is referred to as the external surface and the one facing to the body as the internal surface.

### Data Availability

The datasets generated during and/or analysed during the current study are available at MorphoMuseuM^54^.

## Electronic supplementary material

Supplementary Information
