# Supplementary material for: A new, exceptionally preserved juvenile specimen of Eusaurosphargis dalsassoi (Diapsida) and implications for Mesozoic marine diapsid phylogeny
Source: Sci Rep. 2017 Jun 30;7:4406. doi: 10.1038/s41598-017-04514-x (PMC5493663; doi:10.1038/s41598-017-04514-x)
Supplement: Supplementary file 1 — Supplementary Information [file 41598_2017_4514_MOESM1_ESM.pdf]

Supplementary Information

A new, exceptionally preserved juvenile specimen of *Eusaurosphargis dalsassoi* (Diapsida) and implications for Mesozoic marine diapsid phylogeny

Torsten M. Scheyer[1,*], James. M. Neenan[2], Timea Bodogan[1], Heinz Furrer[1], Christian Obrist[3], Mathieu Plamondon[4]

[1]Paläontologisches Institut und Museum der Universität Zürich, Karl Schmid-Strasse 4, CH-8006 Zurich, Switzerland
[2]Oxford University Museum of Natural History, Parks Road, Oxford, OX1 3PW, UK
[3]Erliackerweg 8, 4462 Rickenbach, BL, Switzerland
[4]Empa, Swiss Federal Laboratories for Materials Science and Technology, Center for X-ray Analytics, Überlandstrasse 129, CH-8600 Dübendorf, Switzerland

Correspondence and requests for materials should be addressed to T.M.S. (tscheyer@pim.uzh.ch).

Content

Supplementary Figures

Supplementary 3D Figure S1 (3D content needs to be enabled in the PDF)

Supplementary 3D Figure S2 (3D content needs to be enabled in the PDF)

Supplementary Figures S3-S12 (no 3D content needed in the PDF)

Supplementary Note

Updated and new scorings for *Eusaurosphargis dalsassoi*

Supplementary References

Supplementary Figure S1. Postcranium of PIMUZ A/III 4380.

Supplementary Figure S2. Skull of PIMUZ A/III 4380.

Supplementary Figure S3. Interpretative reconstruction of the pelvic girdle of *Eusaurosphargis dalsassoi* based on PIMUZ A/III 4380.

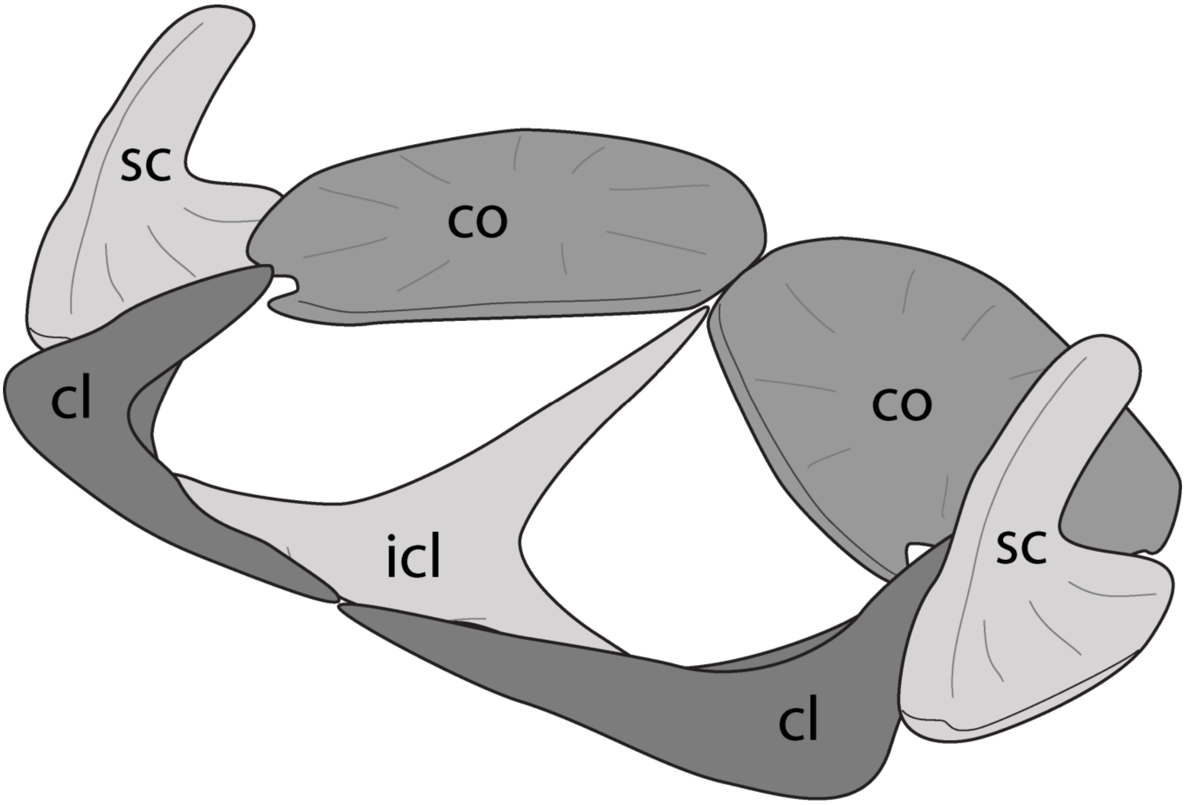

Abbreviations used in the figure are clavicle (cl), coracoid (co), interclavicle (icl), and scapula (sc). Not to scale.

Supplementary Figure S4. Analysis 1 run in TNT.

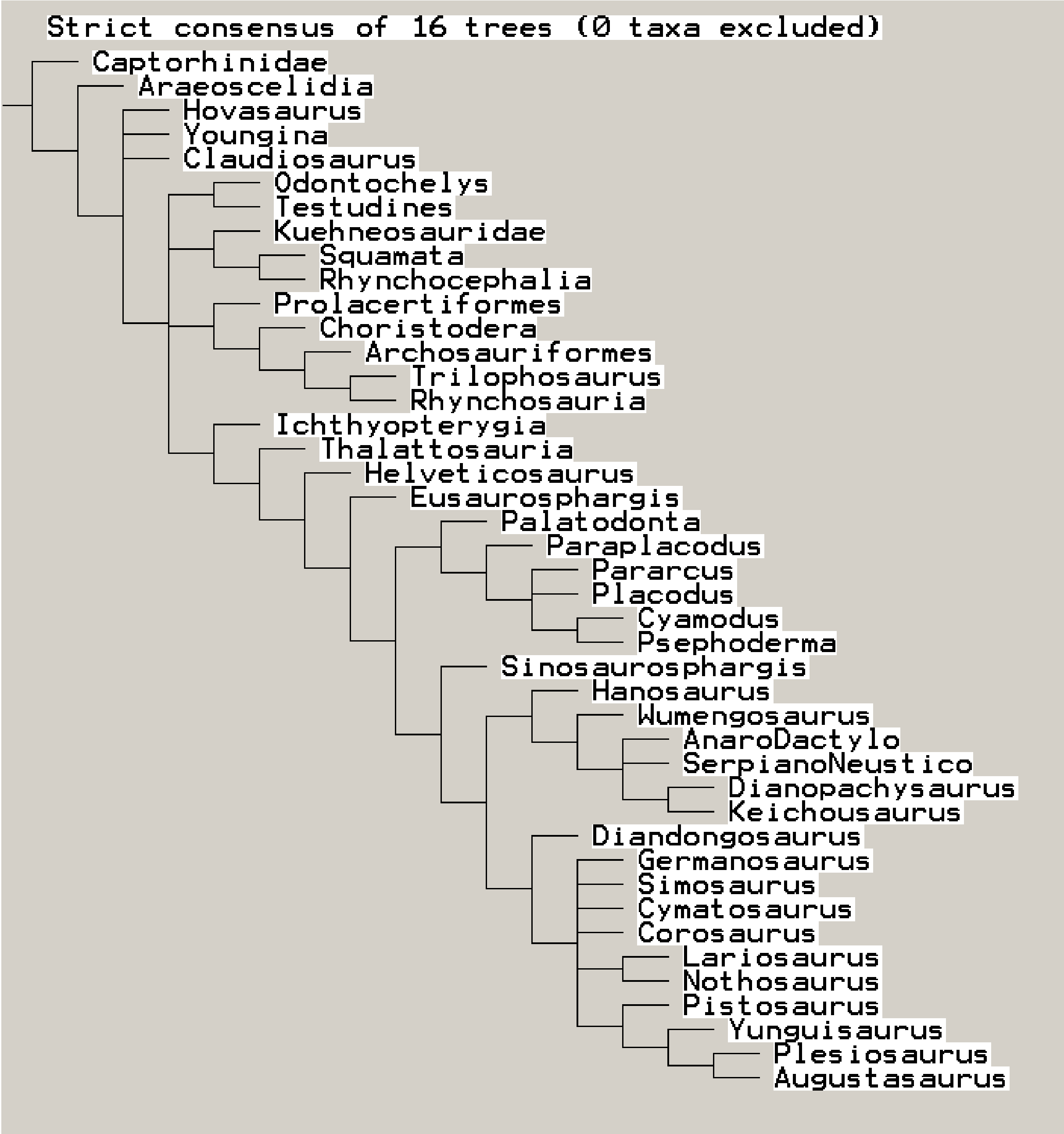

Analysis using the first matrix[1] with all taxa and characters included.

Supplementary Figure S5. Analysis 2 run in TNT.

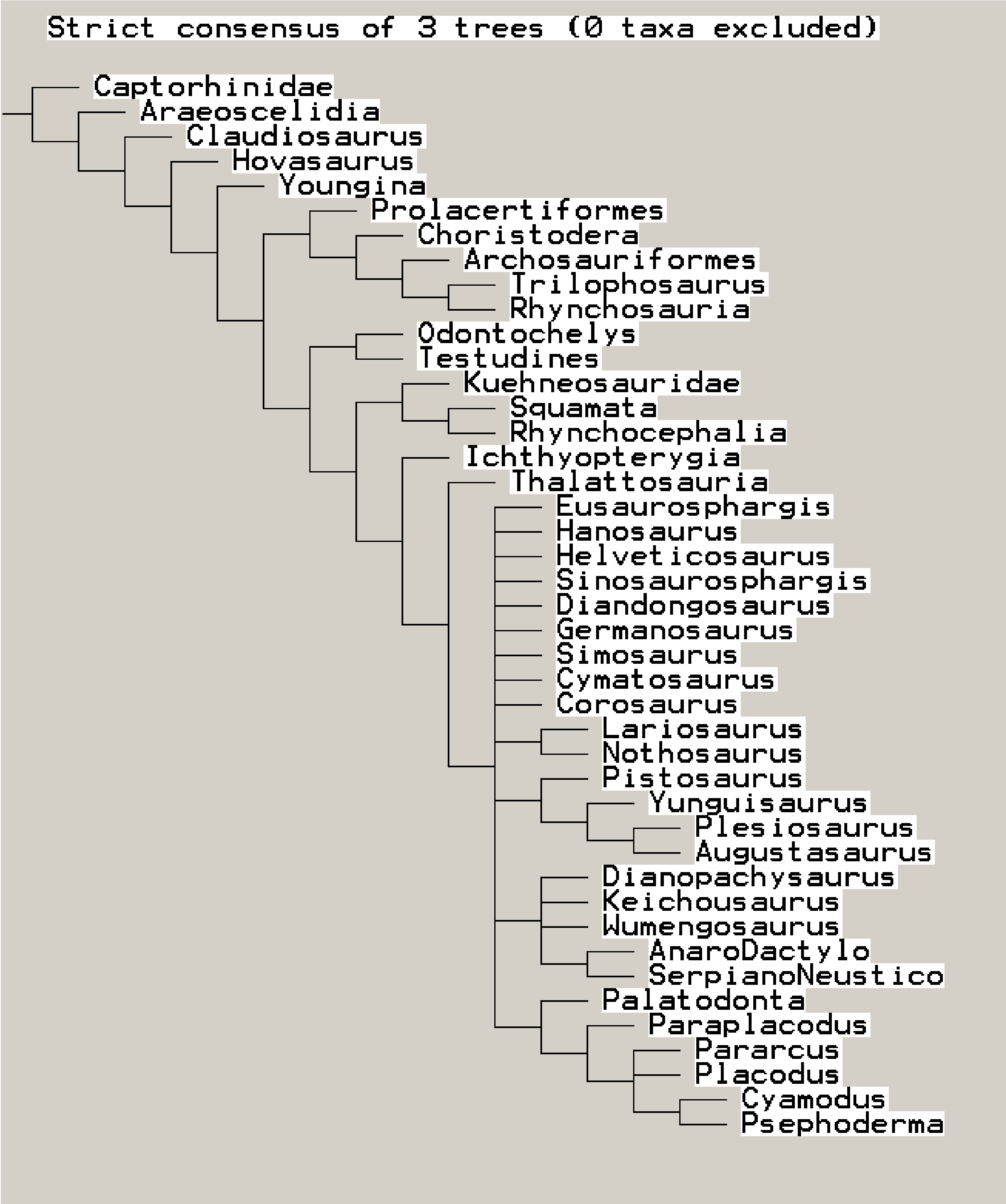

Analysis using the first matrix[1] with all taxa but characters 90, 113, 114, 120, 126, 127, and 131 excluded.

Supplementary Figure S6. Analysis 3 run in TNT.

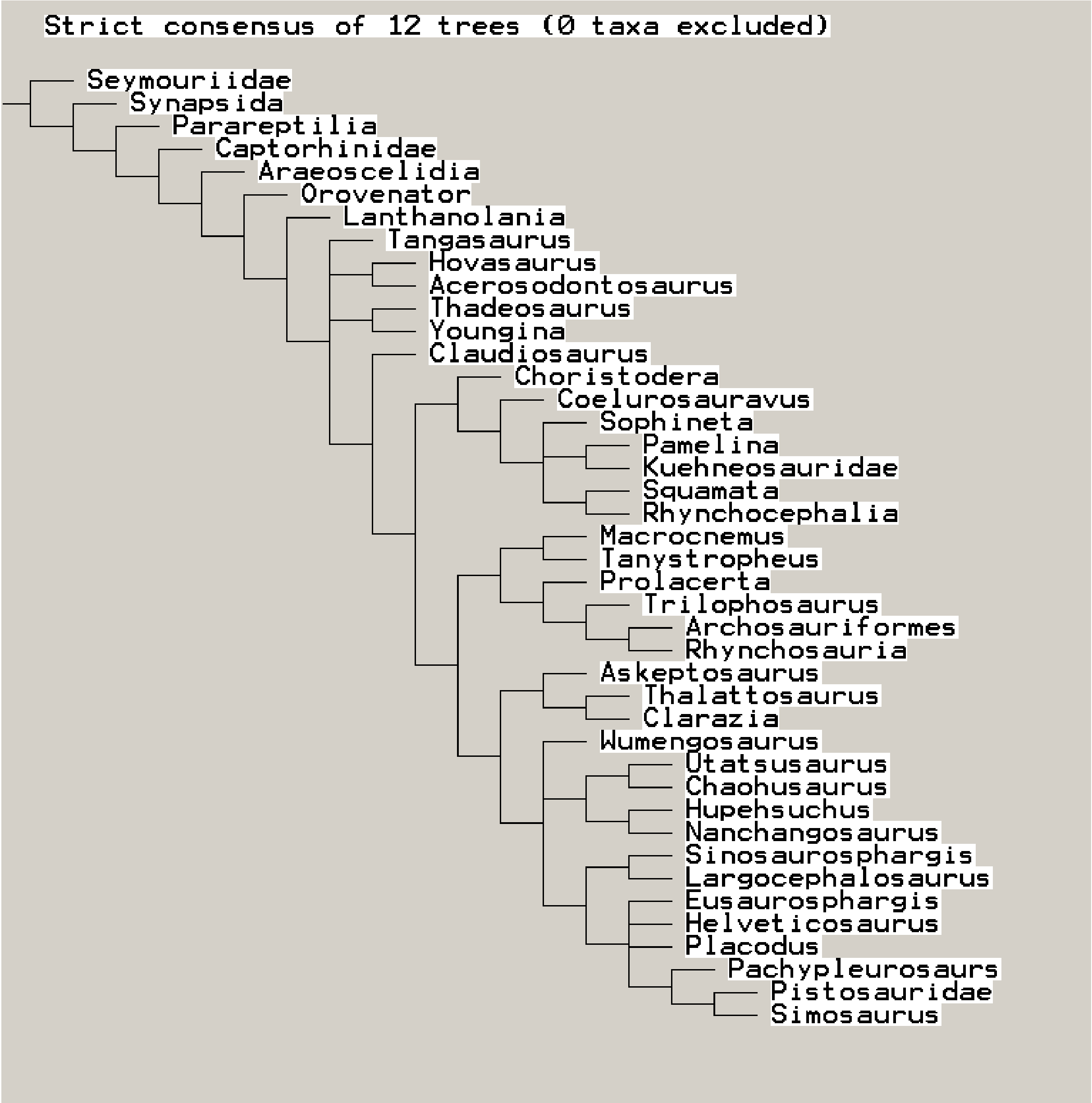

Analysis using the second matrix[2] with with *Eusaurosphargis dalsassoi* added and all characters included.

Supplementary Figure S7. Analysis 4 run in TNT.

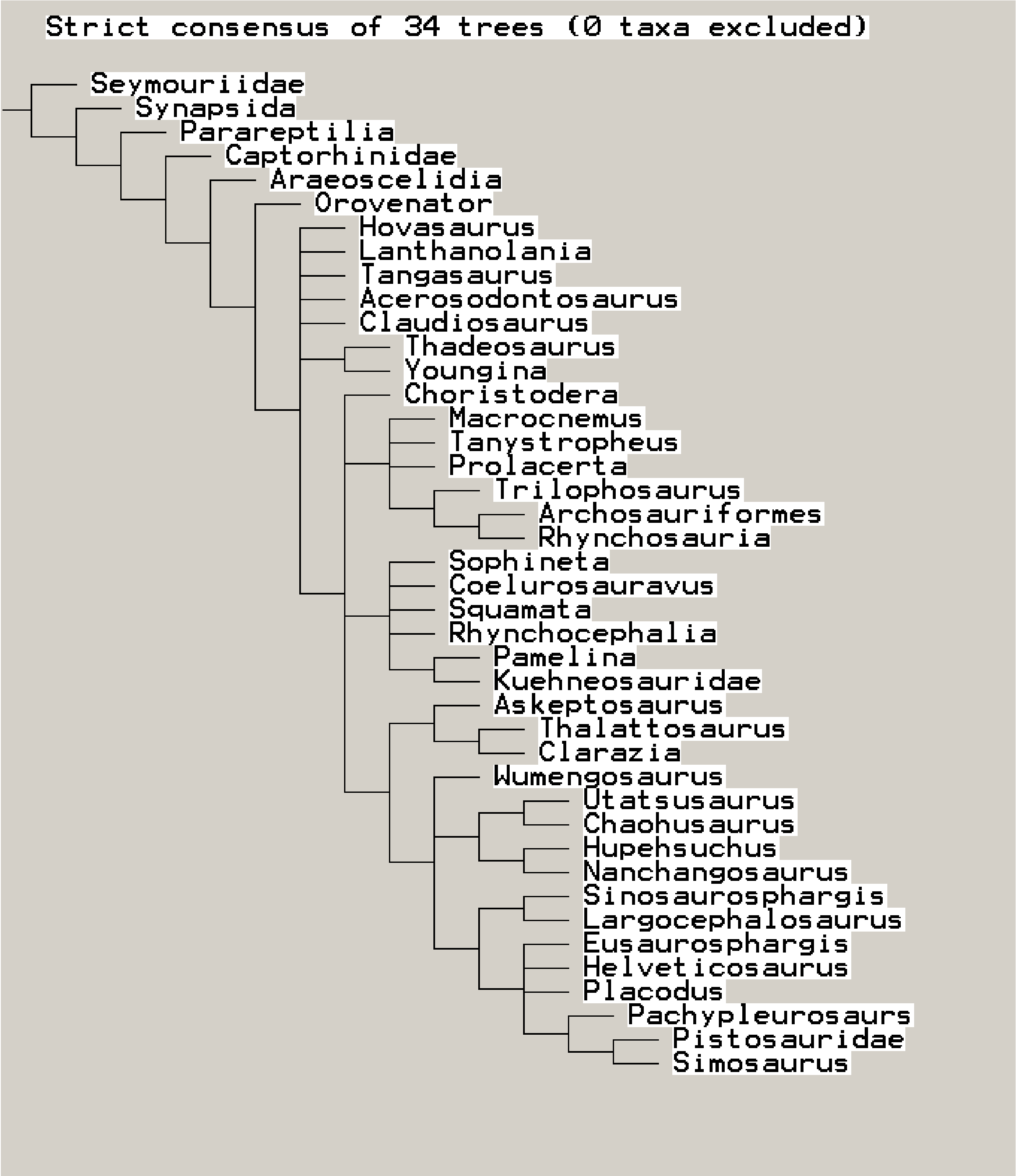

Analysis using the second matrix[2] with *Eusaurosphargis dalsassoi* added, but characters 50, 62, 63, 67, 71, 74, 114, 120, 151, and 171 excluded.

Supplementary Figure S8. Analysis 5 run in TNT.

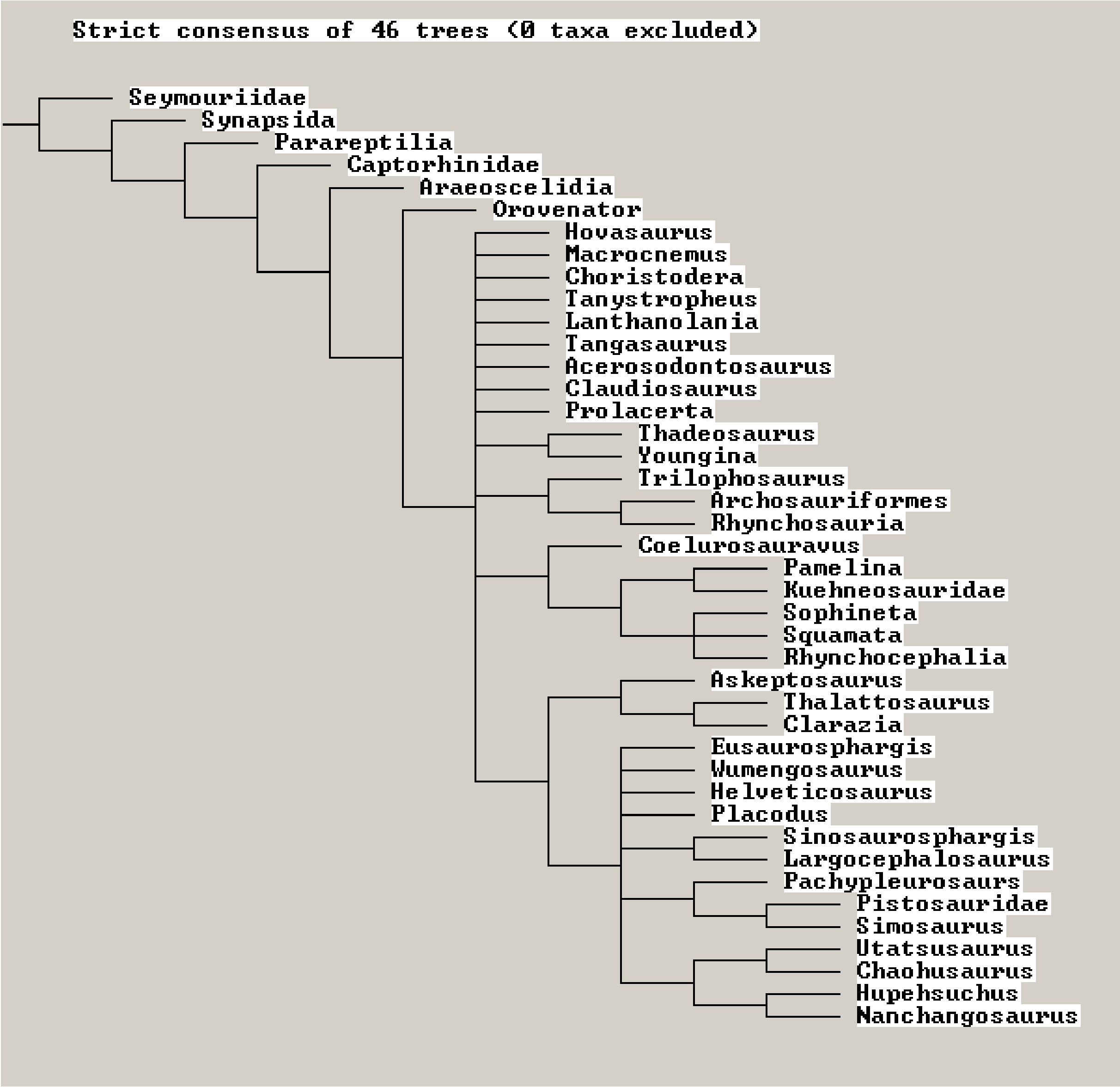

Analysis using the second matrix[2] with *Eusaurosphargis dalsassoi* added, but with characters 1, 9, 50, 58, 61, 62, 63, 67, 68, 71, 74, 114, 120, 145, 151, 171, 180, 186 excluded.

Supplementary Figure S9. Analysis 6 run in TNT.

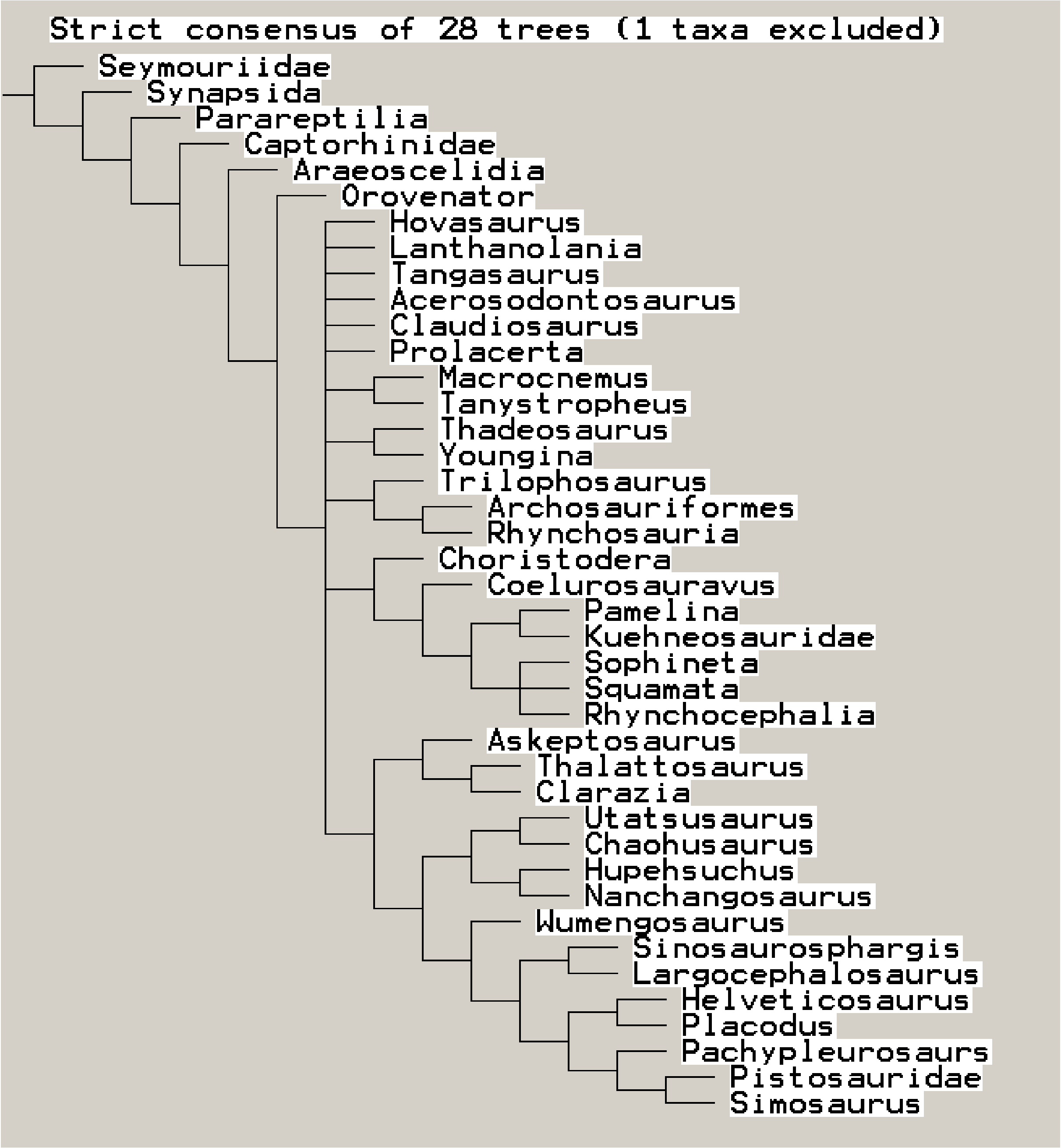

Analysis using the second matrix[2] with *Eusaurosphargis dalsassoi* removed and characters 1, 9, 50, 58, 61, 62, 63, 67, 68, 71, 74, 114, 120, 145, 151, 171, 180, 186 excluded.

Supplementary Figure S10. Analysis 7 run in TNT.

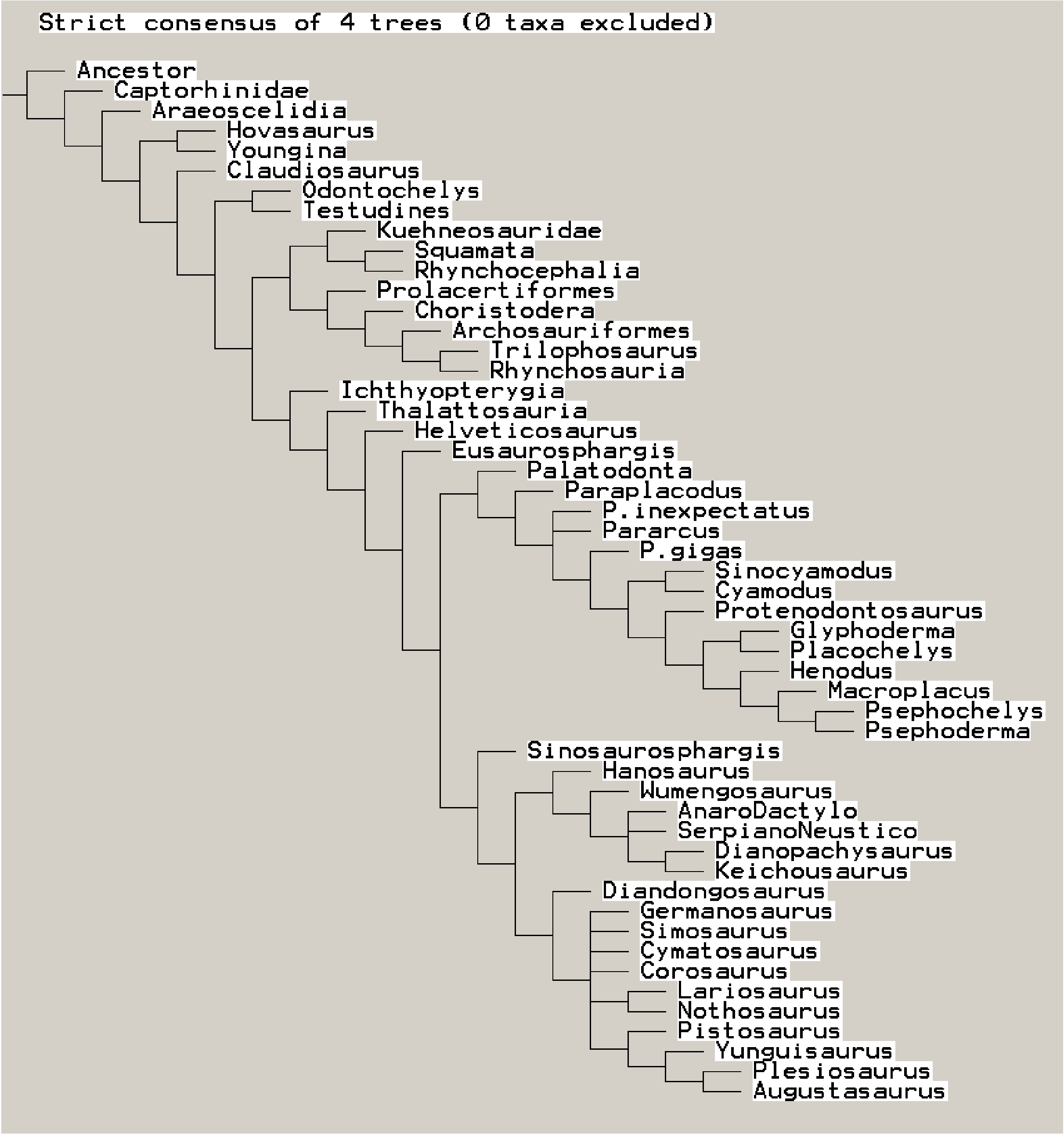

Analysis using the third matrix[3] with ‘all-zero ancestor’ as outgroup.

Supplementary Figure S11. Analysis 8 run in TNT.

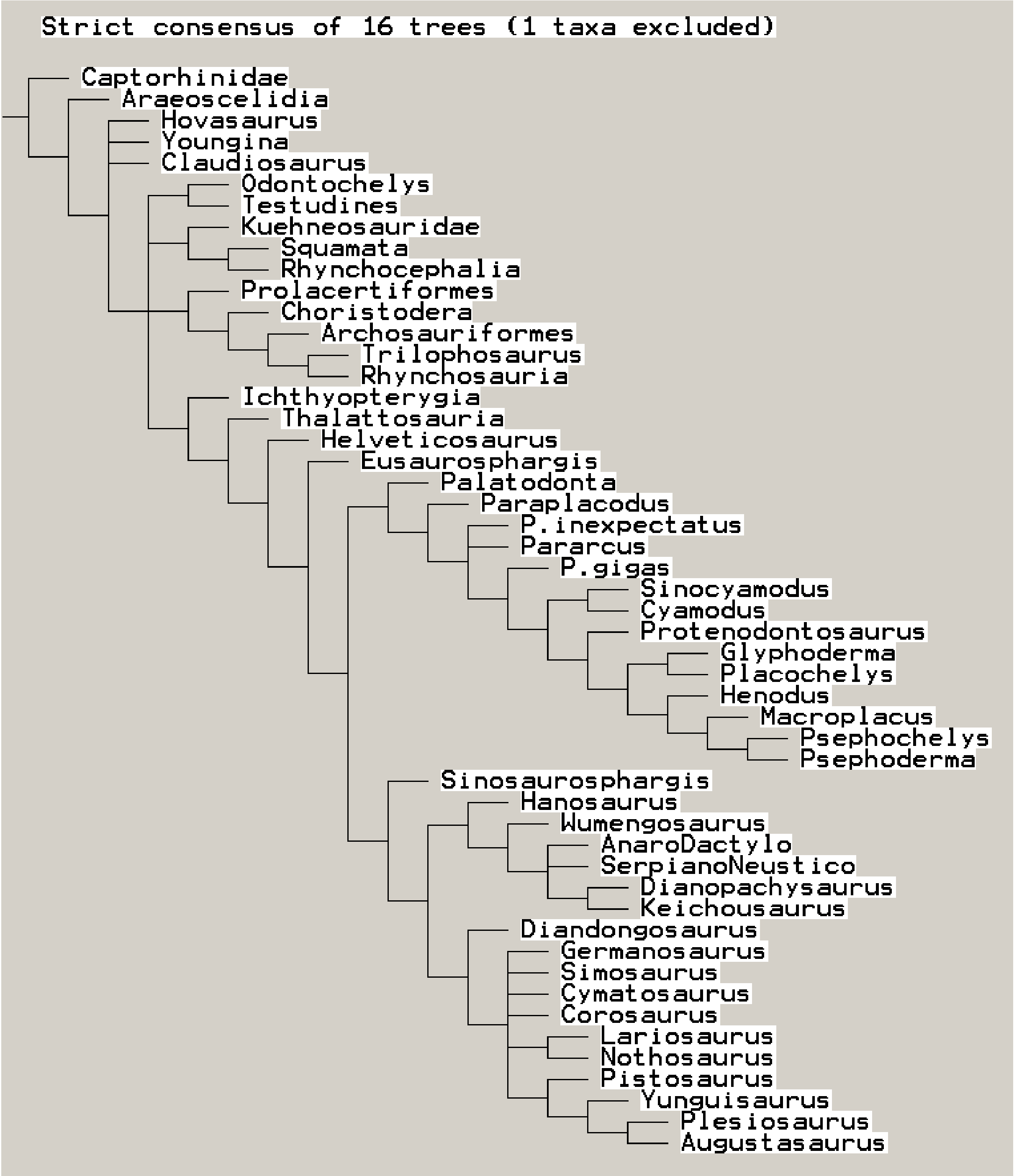

Analysis using the third matrix[3] with ‘all-zero ancestor’ excluded and Captorhinidae as outgroup.

Supplementary Figure S12. Selected postcranial bones of the holotype BES SC 390.

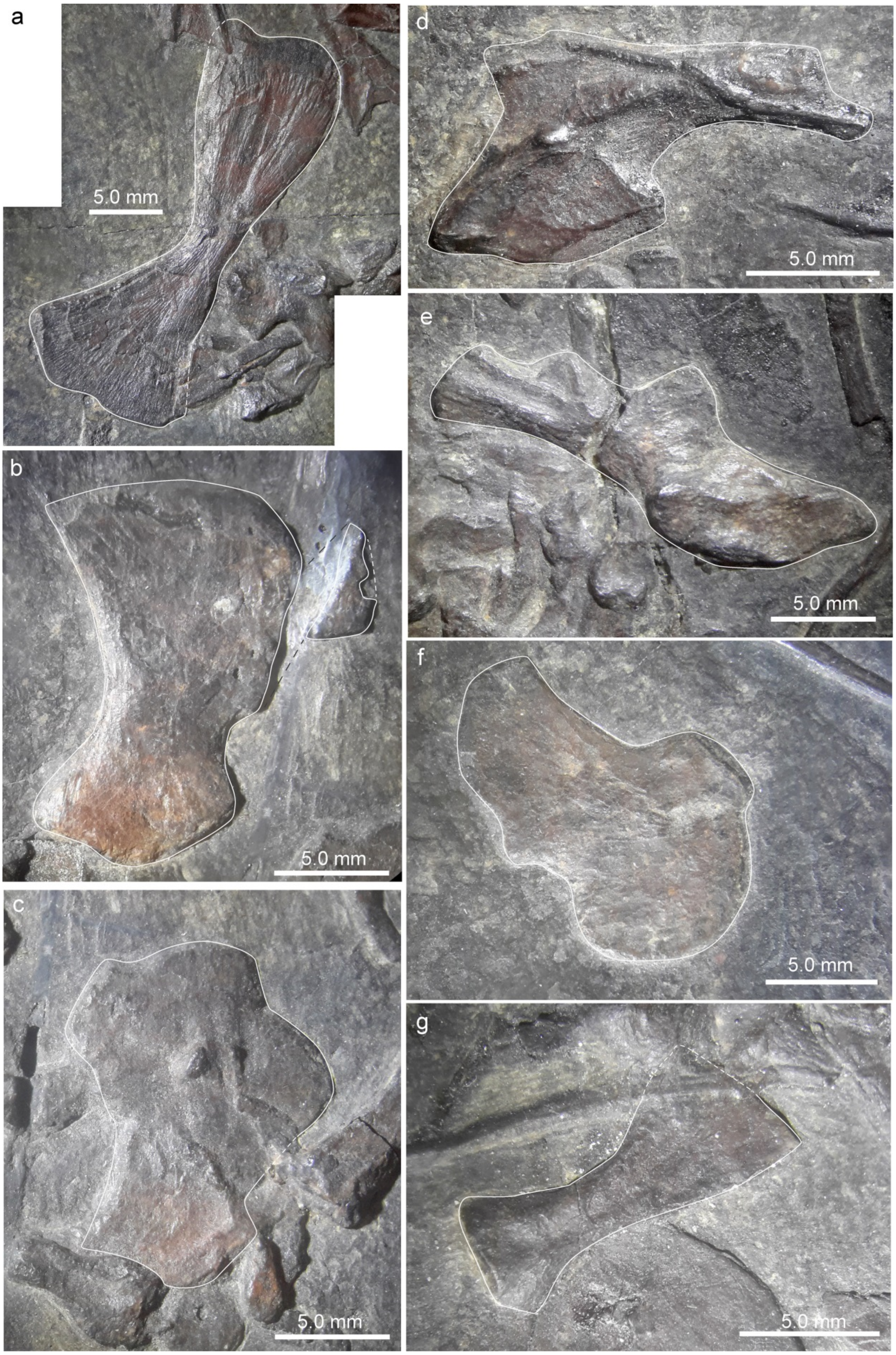

Note that the bone identifications of the original species description[4] are given in parentheses. (**a**) humerus (humerus h_1). (**b**) ischium (pubis p_2); (**c**) ischium (pubis p_1); (**d**) ilium (ilium); (**e**) ilium (unidentified bone); (**f**) pubis (ischium); (**g**) sacral rib 1 (caudal rib car_1).

Supplementary Figure S13. Microanatomy of stylopodial bones of PIMUZ A/III 4380.

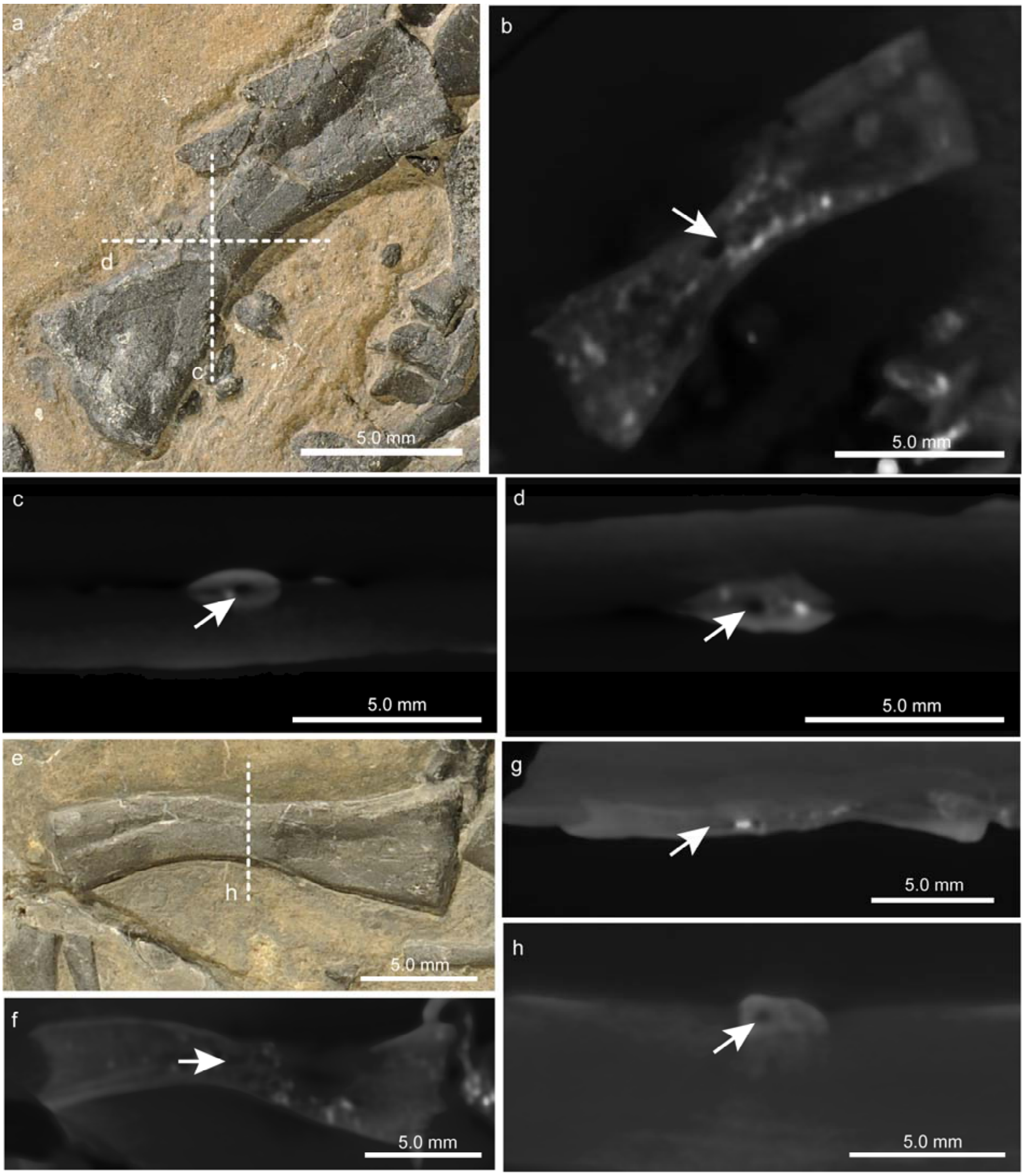

Photograph (**a**) and micro-CT images (**b-d**) of the right humerus, as well as photograph (**e**) and micro-CT images (**f-h**) of the right femur. Note that the position and planes of the cross-sectional slices in c, d, and h are marked by white stipled lines. Medullary cavities are marked by white arrows.

Supplementary Note. Updated and new scorings for *Eusaurosphargis dalsassoi*.

Updated scoring of *Eusaurosphargis dalsassoi* used in Analysis 1 (and Analyses 2 and 3) in the first matrix[1] and in Analysis 7 (and Analysis 8) in the third matrix[3]:

110010???? ?????????? ?003???030 ??0??????? ?0?????1?? 0????1??0? ?000001011 110?000?0? 10?1?10000 ?001110101 00?1001100 0??1?020?0 011?11211? ?10??001?1

New scoring of *Eusaurosphargis dalsassoi* used in Analysis 3 (and Analyses 4 to 6) in the second matrix[2]:

100????13? 010??????? ????0????1 ??????1011 [01]10??1?100 1100001110 ??1?0?01?1 211?0???00 ?00011???? ????????01 ?0?2010?00 0011?00?01 0000?????? 0????????? ????11110? ?0???????? ??2?????11 0010?20??0 ????111000 0?1?[12]1[01]?10 0????00000 ?00

Supplementary References

1 Klein, N. & Scheyer, T. M. A new placodont sauropterygian from the Middle Triassic of the Netherlands. *Acta Palaeontol. Pol.* **59,** 887-902 (2014).

2 Chen, X.-h., Motani, R., Cheng, L., Jiang, D.-y. & Rieppel, O. The enigmatic marine reptile *Nanchangosaurus* from the Lower Triassic of Hubei, China and the phylogenetic affinities of Hupehsuchia. *PlOS ONE* **9(7),** e102361; 10.1371/journal.pone.0102361 (2014).

3 Neenan, J. M., Li, C., Rieppel, O. & Scheyer, T. M. The cranial anatomy of Chinese placodonts and the phylogeny of Placodontia (Diapsida: Sauropterygia). *Zool. J. Linn. Soc.* **175,** 415-428 (2015).

4 Nosotti, S. & Rieppel, O. *Eusaurosphargis dalsassoi* n. gen n. sp., a new, unusual diapsid reptile from the Middle Triassic of Besano (Lombardy, N Italy). *Mem. Soc. ital. sci. nat. Mus. civ. stor. nat. Milano* **31,** 3-33 (2003).
